# MicroRNA miR-30 family regulates non-attachment growth of breast cancer cells

**DOI:** 10.1186/1471-2164-14-139

**Published:** 2013-02-28

**Authors:** Maria Ouzounova, Tri Vuong, Pierre-Benoit Ancey, Mylène Ferrand, Geoffroy Durand, Florence Le-Calvez Kelm, Carlo Croce, Chantal Matar, Zdenko Herceg, Hector Hernandez-Vargas

**Affiliations:** 1 Epigenetics Group. International Agency for Research on Cancer (IARC), 150 rue Albert-Thomas, Lyon, 69008, France; 2 Faculty of Health Sciences, University of Ottawa, Ottawa, K1H 8M5, Canada; 3 Genetic Cancer Susceptibility Group, International Agency for Research on Cancer (IARC), 150 rue Albert-Thomas, Lyon, 69008, France; 4 Ohio State University, 1082 Biomedical Research Tower, 460 W 12th Ave, Columbus, OH 43210, USA; 5 Epigenetics Group, International Agency for Research on Cancer (IARC), 150 cours Albert-Thomas, Lyon cedex 08, 69372, France

**Keywords:** Breast cancer, BT-ICs, Mammospheres, microRNAs, miR-30 family, AVEN

## Abstract

**Background:**

A subset of breast cancer cells displays increased ability to self-renew and reproduce breast cancer heterogeneity. The characterization of these so-called putative breast tumor-initiating cells (BT-ICs) may open the road for novel therapeutic strategies. As microRNAs (miRNAs) control developmental programs in stem cells, BT-ICs may also rely on specific miRNA profiles for their sustained activity. To explore the notion that miRNAs may have a role in sustaining BT-ICs, we performed a comprehensive profiling of miRNA expression in a model of putative BT-ICs enriched by non-attachment growth conditions.

**Results:**

We found breast cancer cells grown under non-attachment conditions display a unique pattern of miRNA expression, highlighted by a marked low expression of miR-30 family members relative to parental cells. We further show that miR-30a regulates non-attachment growth. A target screening revealed that miR-30 family redundantly modulates the expression of apoptosis and proliferation-related genes. At least one of these targets, the anti-apoptotic protein AVEN, was able to partially revert the effect of miR-30a overexpression. Finally, overexpression of miR-30a in vivo was associated with reduced breast tumor progression.

**Conclusions:**

miR30-family regulates the growth of breast cancer cells in non-attachment conditions. This is the first analysis of target prediction in a whole family of microRNAs potentially involved in survival of putative BT-ICs.

## Background

Breast tumor initiating cells (BT-ICs) are functionally defined by their unlimited renewal potential and ability to reproduce tumor heterogeneity, attracting attention as therapeutic targets [1,2]. There is growing evidence that molecular pathways required for normal stem cell functions are deregulated in BT-ICs [3]. As occurs with normal organogenesis and cell differentiation, the selective activation and repression of these pathways may be mediated by microRNAs (miRNAs). These short non-coding RNAs inhibit gene expression by mRNA degradation or translational inhibition [4,5]. Their importance in establishing developmental programs of expression is illustrated by the requirement of miRNA processing proteins like Dicer during embryogenesis [6], and the presence of specific miRNAs in pluripotent cells [7]. Since miRNAs drive terminal differentiation, downregulation of specific miRNAs may play an important role in the development and progression of cancer [8], including breast cancer [9]. Therefore, the aberrant expression of specific miRNAs could lead to a pathologic expansion of immature cells. To gain insight into this untested hypothesis, we performed a miRNA profiling in putative BT-ICs enriched from breast cancer cell lines. Our studies revealed a family of miRNAs that play a key role in defining features of these cells. We further identified and validated the targets of this family of miRNAs and studied its role in survival of BT-ICs in vitro and in vivo.

## Results

### MicroRNA profiling in putative BT-ICs

**Figure 1:**
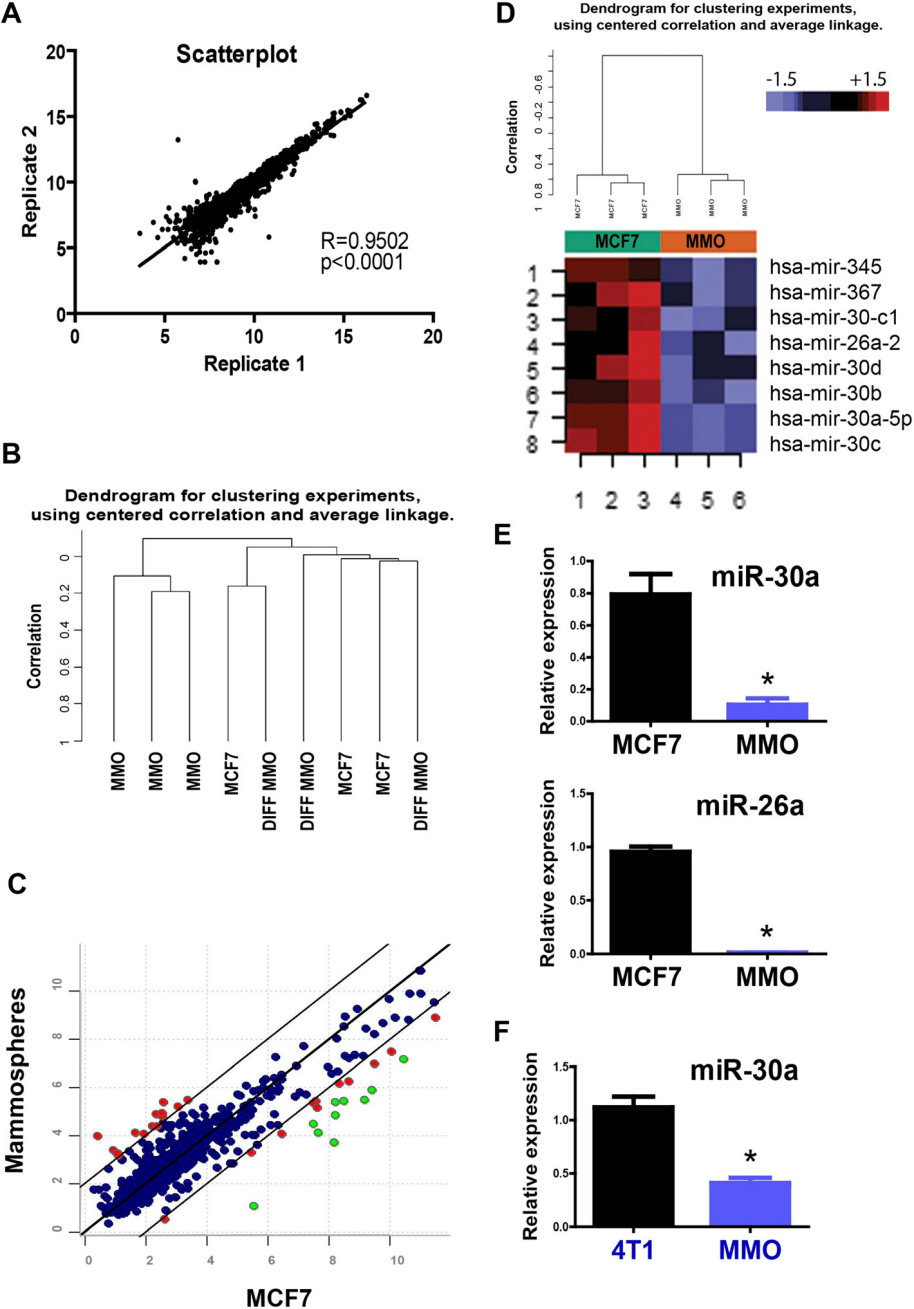
**miRNA profiling in mammospheres.** An oligonucleotide array was used for comparing the miRNA expression between mammospheres (MMO) and parental MCF7 cells. **A**. Scatter plot of 2 technical replicates showing a significant correlation for all miRNA probes. **B**. Unsupervised clustering distinguishing MMO from parental MCF7 cells and from MMO plated in attachment conditions (DIFF MMO). **C**. Scatter plot comparing MMO and MCF7 parental cells. Upregulated and downregulated miRNAs are shown in red. Green dots represent miRNAs significantly downregulated in MMO after class comparison analysis (no miRNA was significantly upregulated in MMO). **D**. Cluster and heat-map analyses are shown for the MMO/MCF7 comparison. miRNAs significantly downregulated in MMO match the miRNAs represented in green in Figure 1C. **E**. Two miRNAs from the previous analysis (miR-30a and miR-26a) were validated using TaqMan probes in independent samples. **F**. miR-30a expression was studied in an unrelated breast cancer cell line, 4T1. Mammospheres derived from 4T1 cells expressed significantly lower levels of miR-30a. P value under 0.05 (two-tailed student t test) is represented with an asterisk (*).

Because of their role in defining expression programs in development and cancer, we investigated whether miRNA expression displayed a particular profile in putative BT-ICs. The non-adherent mammosphere culture system, in which stem-like cells are capable of forming suspended spheres, has been extensively used to enrich cultures for BT-ICs [10]. We used the mammosphere system to compare MCF7 BT-ICs with their parental cell line in a miRNA oligonucleotide array covering 474 human miRNAs. In addition, we plated MCF7-derived mammospheres back to attachment conditions to induce their reconfiguration into an epithelial monolayer (here referred to as “differentiated mammospheres”). Independent biological replicates dis-played high consistency (Figure 1A), while unsupervised clustering discriminated mammospheres from parental MCF7 cells (Figure 1B and Additional file 1: Figure S1A). In contrast, parental MCF7 cells and “differentiated” mammospheres displayed a similar miRNA profile and clustered together after unsupervised analyses, suggesting that mammospheres retained the ability to revert their miRNA expression profile to the original conditions (Figure 1B, and Additional file 1: Figure S1B). Class comparison analyses showed that 8 human miRNAs were differentially expressed between mammospheres and parental MCF7 cells (*p* value <0.001, FDR<0.1), including miR-345, miR-367, miR-26a, and five members of the miR-30 family (Figure 1C, 1D, and Table 1). All these miRNAs were strikingly downregulated in mammospheres (between 8-fold and 22-fold), while their expression increased close to basal levels after plating the mammospheres back to attachment conditions (Additional file 2). When performing a class comparison analysis among the 3 groups (MCF7, mammospheres, and “differentiated” mammospheres), miR-30a-5p displayed the most consistent capacity to distinguish mammospheres from the other two groups (lowest p and FDR values).

**Table 1:** miRNAs differentially expressed in mammospheres

**Symbol**	**MCF7**	**MMO**	**Fold-change**
**hsa-mir-30c**	670.5	59.69	11.23
**hsa-mir-30b**	566.7	44.93	12.61
**hsa-mir-30c-1**	289.3	13.11	22.07
**hsa-mir-367**	45.6	2.13	21.37
**hsa-mir-30a-5p**	355.7	43.7	8.14
**hsa-mir-30d**	1393	142.04	9.81
**hsa-mir-26a-2**	201.2	17.16	11.73
**hsa-mir-345**	178.1	22.34	7.97

Mean expression values and fold-change (MCF7/MMO) are shown for MCF7 cells and MCF7-derived mammospheres (MMO). P-value < 0.001, FDR < 0.1.

No miRNAs were significantly overexpressed in mam-mospheres, and therefore we focused our attention in those miRNAs significantly downregulated. Results were validated using an independent expression array platform, together with specific Taqman qRT-PCR assays. Results obtained with the Illumina Human v2 bead array, were consistent with the oligonucleotide array data, showing no significantly overexpressed miRNAs in mammospheres (Additional file 3: Figure S2A-C). miR-30a was the most significantly down regulated miRNA in mammospheres compared to parental MCF7 cells, while miR-26a and miR-345 were also found to be significantly downregulated (Additional file 3: Figure S2D). The differential expression of several miRNAs including miR-30a and miR-26a were further confirmed using TaqMan probes (Figure 1E). Absolute copy number quantification was performed by using a standard miR30a probe at different dilutions (Additional file 4: Figure S3A and Figure S3B). Extrapolating to these standards, we defined an average of approximately 20 copies of miR-30a per MCF7 cell. This is significantly higher than the 1 copy per cell obtained in mammospheres (Additional file 3: Figure S2B). In addition, a significant down-regulation of miR-30a expression was found in mammospheres derived from the non-related mammary cancer cell line, 4T1, relative to parental 4T1 cells (Figure 1F). These results have revealed a panel of differentially expressed miRNAs, and demonstrated that miR-30 family downregulation is not cell line specific, and may indeed play an important role in mammosphere formation and maintenance of cell growth under non-attachment conditions.

### miR-30a regulates non-attachment growth in putative BT-ICs

**Figure 2:**
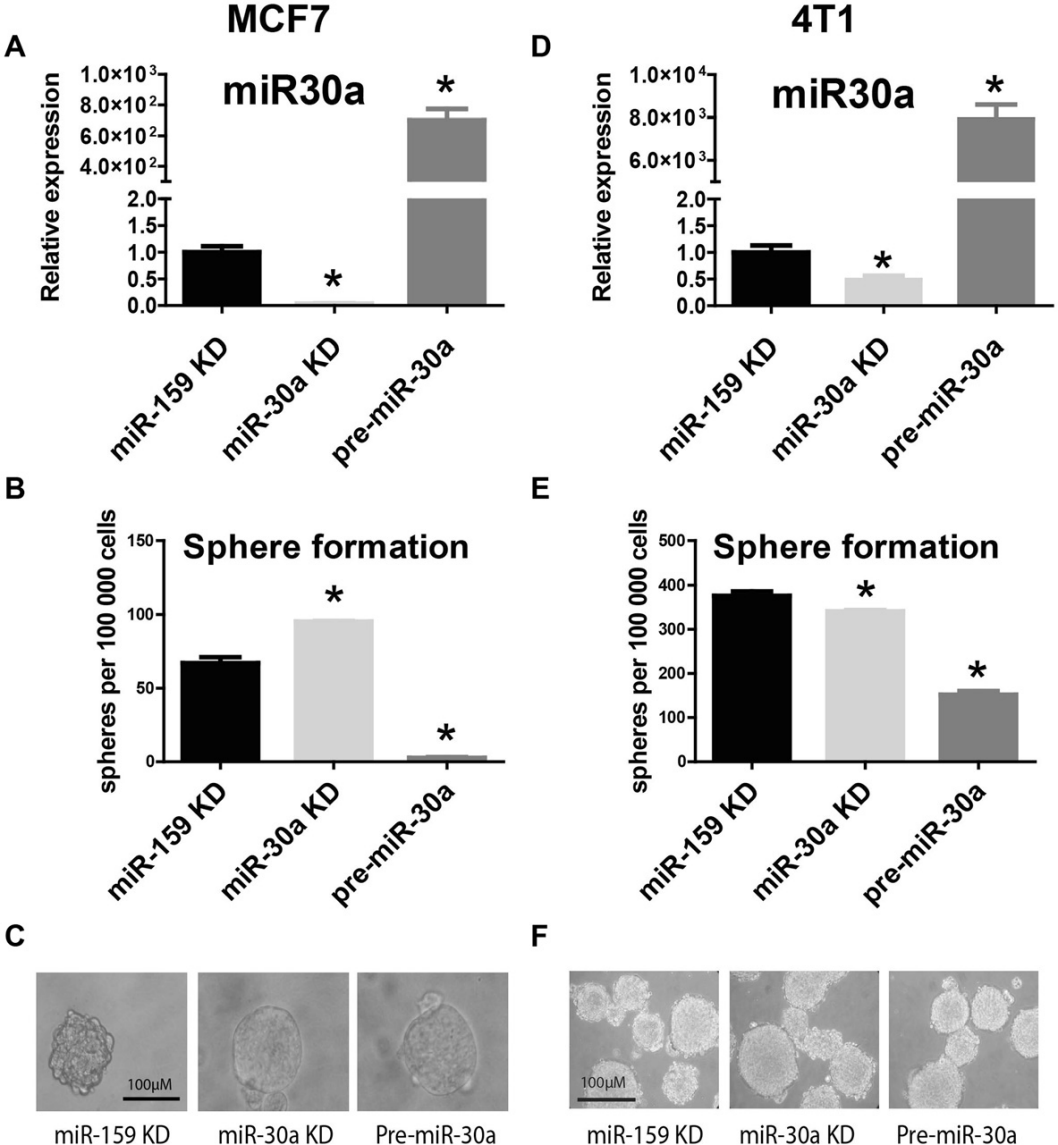
**Sphere formation assay after modulation of miR-30a expression.** MCF7 and 4T1 breast cancer cells were transiently transfected with miR-30a knock-down (KD), Pre-miR-30a, and miR-159 KD control probes. Efficiency of transfection was verified by TaqMan qRT-PCR (**A**, **D**). 48 hours following transfection, cells were used in a mammosphere assay (**B**, **E**). MMO were counted after 4 days. Technical triplicates were used for statistical analyses, with one representative experiment shown (**C**, **F**). P values (for comparisons to control miR-159 KD) under 0.05 (two-tailed student t test) are represented with an asterisk (*).

Among differentially expressed miRNAs in mam-mospheres, miR30a-5p (referred to here and thereafter as miR30a) displayed the most consistent (across all platforms) and significant downregulation (lowest p value). Therefore, we chose to address the functional role of this miRNA in putative BT-ICs. We experimentally modulated miR-30a levels and studied the capacity to form mammospheres in vitro, as an extensively used assay to estimate the capacity of self-renewal and proliferation [10-12]. To this end, MCF7 breast cancer cells were transfected with either miR-30a inhibitor (KD) oligos (to suppress its expression), or pre-miR-30a precursor oligos (to overexpress miR-30a) during 48 hours, and studied cellular response to downregulation and overexpression of miR30a. As a control, cells were also transfected with miR-159 inhibitor (KD) oligos, a miRNA known to lack targets in the human genome [13] (Figure 2A and Additional file 4: Figure S3C). We found a dramatic reduction in the number of mammospheres formed after over-expression of miR-30a in MCF7 cells (mean 2.66 spheres/well compared to more than 50 spheres/well in control miR-159-KD, p<0.01) (Figure 2B). In contrast, miR-30a KD transfection significantly enhanced the formation of mammospheres in MCF7 cells (Figure 2B). Inhibition or overexpression of miR-30a did not affect mammospheres morphology or size (Figure 2C).

To further test the generality of the impact of miR-30a regulation in mammosphere formation, we transfected an independent breast cancer cell line (4T1) with miR-30a KD and pre-miR-30a precursor oligos and examined its ability to grow in non-attachment conditions. In general, 4T1 cells displayed an enhanced ability to produced mammospheres compared to MCF7 cells, consistent with the higher invasiveness and metastatic potential of these cells. Importantly, also in 4T1 cells, transfection with pre-miR-30a resulted in a striking reduction in 4T1-derived mammosphere formation (mean 152 spheres/well compared to almost 400 spheres/well in control miR-159-KD, p<0.01) (Figure 2D-2F), consistent with the results obtained in MCF7 cells. However, in contrast to MCF7 cells, we observed a slight (although statistically significant) reduction in the number of mammospheres after downregulation of miR-30a (mean 341, p=0.03) (Figure 2E). Of note, transfections did not have any effect in cell growth and viability of parental 4T1 and MCF7 cells. Together, these results revealed a functional role of miR-30a in sustaining the growth of breast cancer cells in non-attachment conditions and suggest that miR-30a may regulate essential pathways for the self-renewal of putative BT-ICs.

### Identification of miR-30a target genes in putative BT-ICs

**Figure 3:**
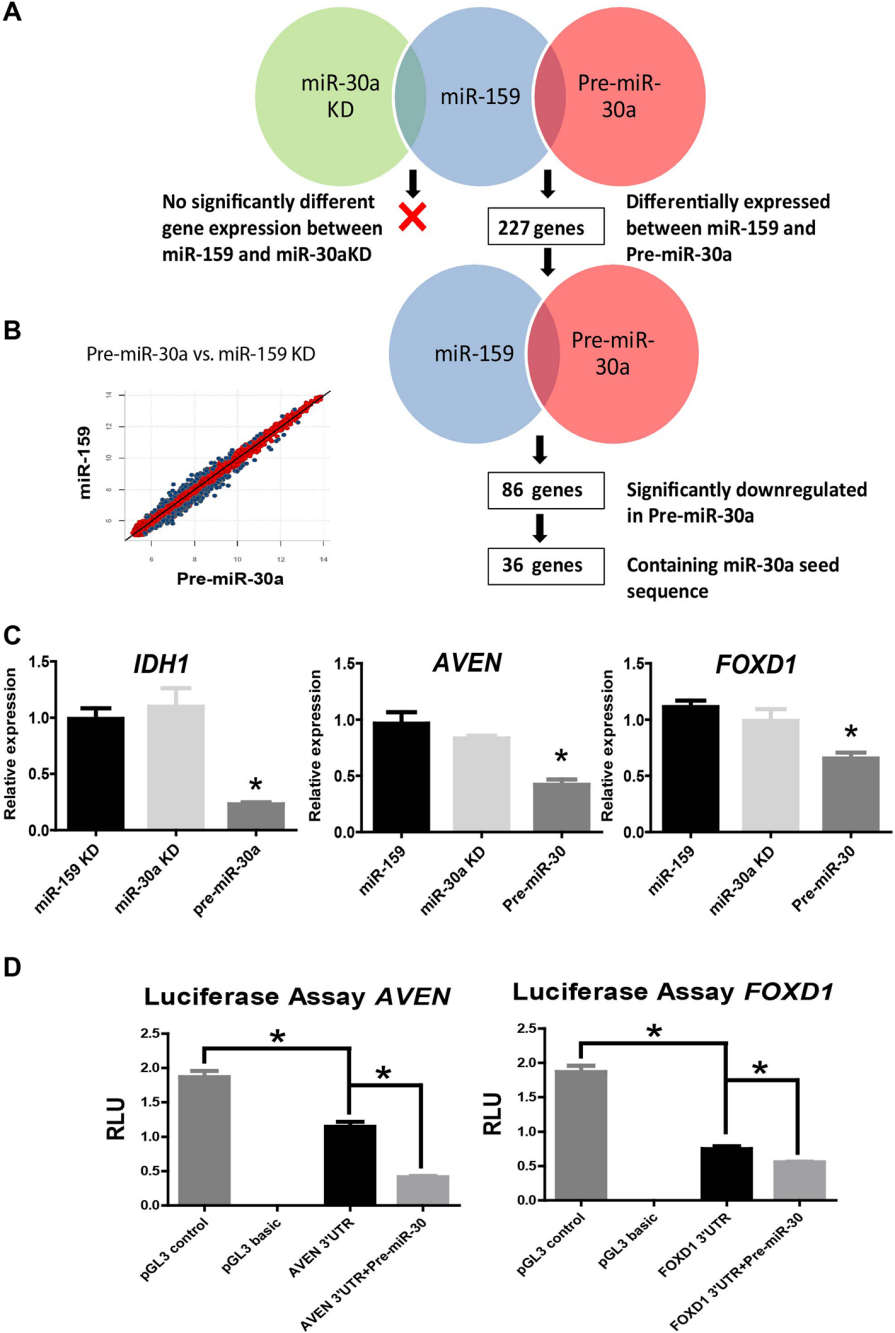
**Whole genome expression after modulation of miR-30a expression.** Same transfections described in Figure 2 were done before transcriptome analyses. **A**. Strategy for identification of potential miR-30a targets. As shown, no significant differences were found between miR-30a-KD and control miR-159-KD cells. Therefore, downstream analyses focused on the Pre-miR-30a conditions. **B**. Scatter plot comparing Pre-miR-30a to miR-159-KD control condition. From 227 significant genes in this comparison, 86 were downregulated, 36 of which contained a miR-30a seed sequence. **C**. Validation of a subset of these 36 genes was done with qRT-PCR (see also Additional file 4: Figure S3). Gene expression values are relative to HPRT1 housekeeping gene. P values (for comparisons to control miR-159 KD) under 0.05 are represented with an asterisk (*). **D**. Target validation was done after cloning 3^′^UTR sequences of AVEN and FOXD1 into a luciferase reporter plasmid and transfection of MCF7 cells. P value under 0.05 (two-tailed student t test) is represented with a (*).

miRNAs are able to regulate their target genes by decreasing their mRNA levels [14]. Therefore, we screened for miR-30a targets using whole genome expression bead arrays after transfecting MCF7 cells with miR-30a-KD probe and miR-30a precursor, as well as miR-159-KD (control) oligos (Figure 3A). This assay produced high quality data with strong correlation between biological replicates (Additional file 5: Figure S4A). Although unsupervised clustering was able to clearly distinguish MCF7 cells overexpressing miR-30a, samples from cells inhibited for miR-30a clustered together with control samples (Additional file 5: Figure S4B). Consistent with this finding, although 227 genes were differentially expressed between pre-miR-30a-transfected and control cells, our analysis showed no differentially expressed genes between the miR-30 KD and control cells (Figure 3A). As miR-30 KD oligos had a significant effect on sphere formation (Figure 2B), this result indicates that biologically significant targets may fall below the sensitivity of the assay or the thresholds used for the analyses (as discussed below). Among 227 differentially expressed genes in pre-miR-30a transfected cells, there were 86 genes downregulated, suggesting that these may be direct targets of miR-30a (Figure 3B). The miRNA seed sequence serves to direct the miRNA to its mRNA targets [15]. Therefore, to identify the genes that are likely to be bona fide targets of miR-30a, we took advantage of publicly available algorithms to identify the genes with 3^′^UTR region containing miR-30a seed sequences (Table 2 and Additional file 2). Our analysis identified 36 potential targets, some of which (such as *SEC23A*) have been previously reported [5]. For validation, we chose a subset of differentially expressed genes (including the apoptosis-related gene *AVEN*, the transcription factor-related genes *FOXD1* and *TFDP1*, and previously validated targets such as *IDH1* and *SEC23A)*. In all cases, qRT-PCR confirmed the bead array results using independent biological replicates (Figure 3C and S4C). Importantly, this validation confirmed the downregulation of potential miR-30a targets after miR-30a overexpression, whereas no difference in gene expression between miR-30a-KD and miR-159-KD control transfected cells was found (Figure 3C and Additional file 5: Figure S4C), consistent with the bead array transcriptome data.

**Table 2:** Putative targets of miR-30a after whole genome expression (WGE) analysis of miR-30a overexpression

**Gene name**	**Pre-miR-30a**	**miR-159 k.d.**	**Fold-change**	**Score**
***ACP2***	103.98	160.67	0.65	−6.23
***ARID5B***	97.55	145.53	0.67	−2.96
***ARVCF***	44.57	61.42	0.73	−2.66
***ATG12***	134.92	192.78	0.70	−3.36
***AVEN***	89.8	166.93	0.54	−9.51
***BNIP3L***	64.7	95.14	0.68	−4.93
***C14orf129***	165.21	278.97	0.59	−1.92
***C1orf19***	291.73	475.23	0.61	−11.7
***C2orf30***	175.86	317.2	0.55	−10.18
***C3orf57***	855.03	1500.97	0.57	−1.39
***CHMP2B***	294.63	447.1	0.66	−7.54
***DCUN1D3***	85.59	141.19	0.61	1.38
***DPY19L1***	194.24	277.5	0.70	−4.87
***DPYSL2***	315.4	502.3	0.63	−5.31
***EDG3***	100.43	149.23	0.67	−1.99
***ELMOD2***	81.24	123.28	0.66	1.74
***FAM18B***	121.66	169.09	0.72	−4.05
***FOXD1***	104.98	150.49	0.70	−2.49
***GNAI2***	229.93	496.33	0.46	−11.2
***GNG10***	91.32	139	0.66	−7.76
***GNPDA1***	489.02	831.37	0.59	−0.6
***KDELC2***	116.08	204.6	0.57	−3.39
***KIAA0241***	99.59	151.45	0.66	−2.69
***NECAP1***	157.52	253.56	0.62	−6.58
***PDSS1***	257.51	381.04	0.68	5.63
***PGM1***	196.55	309.58	0.63	−6.5
***PIK3R2***	762.04	1060.6	0.72	−5.76
***PPP1R2***	279.79	504.81	0.55	−9.54
***REEP3***	56.69	79.57	0.71	−5.93
***REXO4***	174.68	257.94	0.68	−6.87
***RPA2***	385.11	641.74	0.60	−1.52
***SEC23A***	54.91	75.17	0.73	−4.48
***TFDP1***	709.63	1059.53	0.67	−7.84
***TM4SF1***	436.61	828.33	0.53	−6.84
***TRIB3***	277.11	422.9	0.66	−2.06
***VKORC1L1***	335.84	525.07	0.64	−3.44

Significantly downregulated genes after Pre-miR-30a transfection. Only potential targets with 3′UTR seed sequences of miR-30a are included. “Scores” are based on the PITA algorithm, as described in Materials and Methods. Targets common to miR-30 family WGE analysis are underlined.

Among the significantly downregulated genes, we selected *FOXD1* and *AVEN* for further validation using luciferase assays. *AVEN* was amongst the most significantly downregulated genes after miR-30a over-expression, while *FOXD1* was a predicted miR-30a target by 6 different algorithms (miRanda, PicTar, PITA, TargetScan, RNAhybrid, and MiRTarget2). We cloned the 3^′^UTR sequences of *FOXD1* and *AVEN,* containing the seed sequence of miR-30a, in pGL3 control vector expressing constitutively the luciferase gene. A construct containing the 3^′^UTR sequence lacking the complete miR-30a seed sequence was used as a control. These constructs were transfected into MCF7 cells, which express low, but detectable, levels of endogenous miR-30a (Figure 2 and Additional file 4: Figure S3). A significant reduction in luciferase expression was observed after transfection with both 3^′^UTR constructs, compared to pGL3 control (Figure 3D). Moreover, a further reduction in luciferase activity was observed after overexpressing miR-30a in co-transfected cells (Figure 3D). These results suggest that miR-30a is specifically targeting the 3^′^UTR regions of *FOXD1* and *AVEN*. Together, these findings support the differentially expressed genes as direct targets of miR-30a and thus they may play a major role in mammosphere growth. However, a potential compensatory effect by other members of the miR-30 family may explain the lack of effect after efficient downregulation of miR-30a.

### miR-30 family displays gene target redundancy in breast cancer cells

**Figure 4:**
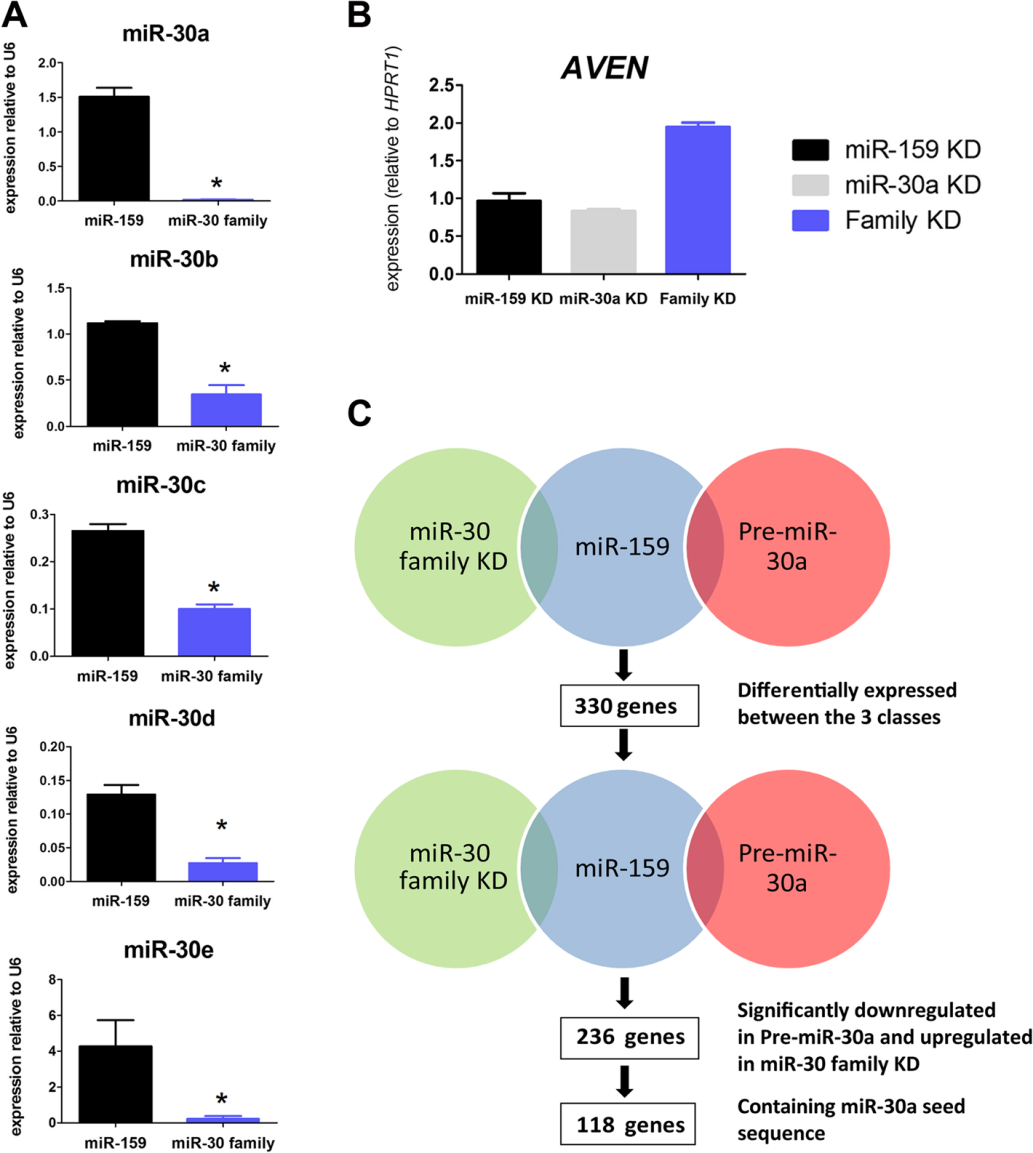
**Whole genome expression after modulation of miR-30 family expression.** miR30 family knock-down was obtained by using 2 oligonucleotides, as described in Methods. **A**. Efficiency of KD in MCF7 cells was checked by TaqMan qRT-PCR for five members of the miR-30 family. **B**. a potential new target of miR-30a, *AVEN*, was also checked. **C**. Strategy for identification of potential miR-30a targets. As opposed to Figure 3A, class comparison analysis was able to show 330 miRNAs differentially expressed between the 3 classes. 236 genes were commonly upregulated after miR-30 family KD and downregulated after miR-30a overexpression. Out of these, 118 genes contained a miR-30a seed sequence. P value under 0.05 (two-tailed student t test) is represented with a (*).

Although the large majority of miRNA targets remain unknown, there is evidence for redundant target specificity of unrelated miRNAs, or miRNAs from the same family [16,17]. Our data on miR-30a knocked-down cells suggested a compensatory effect by other member of the miR-30 family. Therefore, to test for target redundancy within the miR-30 family, we used a custom-designed probe-set to simultaneously inhibit all 5 members of the family: miR-30a, b, c, d and e. A significant downregulation of all miR30 microRNAs was observed after transfection with family inhibitor probe-set, confirming the global effect of the family inhibitor (Figure 4A). Moreover, downregulation of the miR-30 family correlated with overexpression of one of the new potential target genes, *AVEN,* an effect not observed when only miR-30a was inhibited (Figure 4B). Next, we performed a new transcriptome analysis on MCF7 cells transfected with miR-30 family KD probes, miR-30 precursor or miR-159 KD (control). In contrast to the first whole genome expression analysis, a three class comparison revealed this time 330 differentially expressed genes distinguishing the three classes (p<0.001, FDR<0.15). We next looked for those genes simultaneously upregulated after KD and downregulated after precursor transfection, as they were most likely to be direct targets (Figure 4C). We found 236 genes that overlapped between these two conditions. Out of these, 118 genes were found to have a 3^′^UTR region containing miR-30a seeds. Importantly, this analysis revealed that among the genes whose expression is most strongly influenced by miR-30 family KD were *IDH1*, *AVEN* and *FOXD1*, all of which had been identified in the previous transcriptome analysis (Table 2). Indeed, out of 36 targets of miR30a identified in the first screening, 26 (72%) were also identified after silencing miR30 family in the second screening. Moreover, this last screening included 8 experimentally validated targets of miR-30 [5], such as *P4HA2* and *CBFB*, that had not been revealed by the previous whole genome expression analysis. These results suggest that miR-30 family members display target redundancy, and the downregulation of all of its members is necessary for growth under non-attachment conditions.

To define functional enrichment of miR30 targets, we used the Database for Annotation, Visualization and Integrated Discovery (DAVID ) v6.7. First, we used the final list of 118 putative targets of miR30 family of miRNAs. However, no significant enrichment was found for any functional category or pathway when using the whole human transcriptome as a reference. Next, we performed enrichment analyses in the extended 330 genes list, that represents all transcripts significantly regulated (in any direction) after miR30 family modulation, independently of the presence of a putative seed sequences in their 3^′^UTR. We analyzed their enrichment in specific Gene Ontology (GO) categories, and biological pathways (BioCarta and KEGG). Only those categories with p value < 0.005 for three different statistical tests (LS and KS permutation, and Efron-Tibshirani’s GSA test), were considered as statistically significant. Top significant GO category was cytokinesis, while both GO and BioCarta pathway analyses were significant for a number of metabolic processes (e.g. cellular aldehyde, acetyl-CoA, reductive carboxylate cycle, and galactose metabolism) (Additional file 6). Interestingly, KEGG pathway analysis produced two significant categories: Polyadenylation of mRNA, and EGF Signaling Pathway. Polyadenylation is known to be involved in mRNA stability, while EGF signalling has been shown to promote cancer cell proliferation and to enhance mammosphere formation [18].

### miR-30 overexpression impairs breast cancer tumor formation

**Figure 5:**
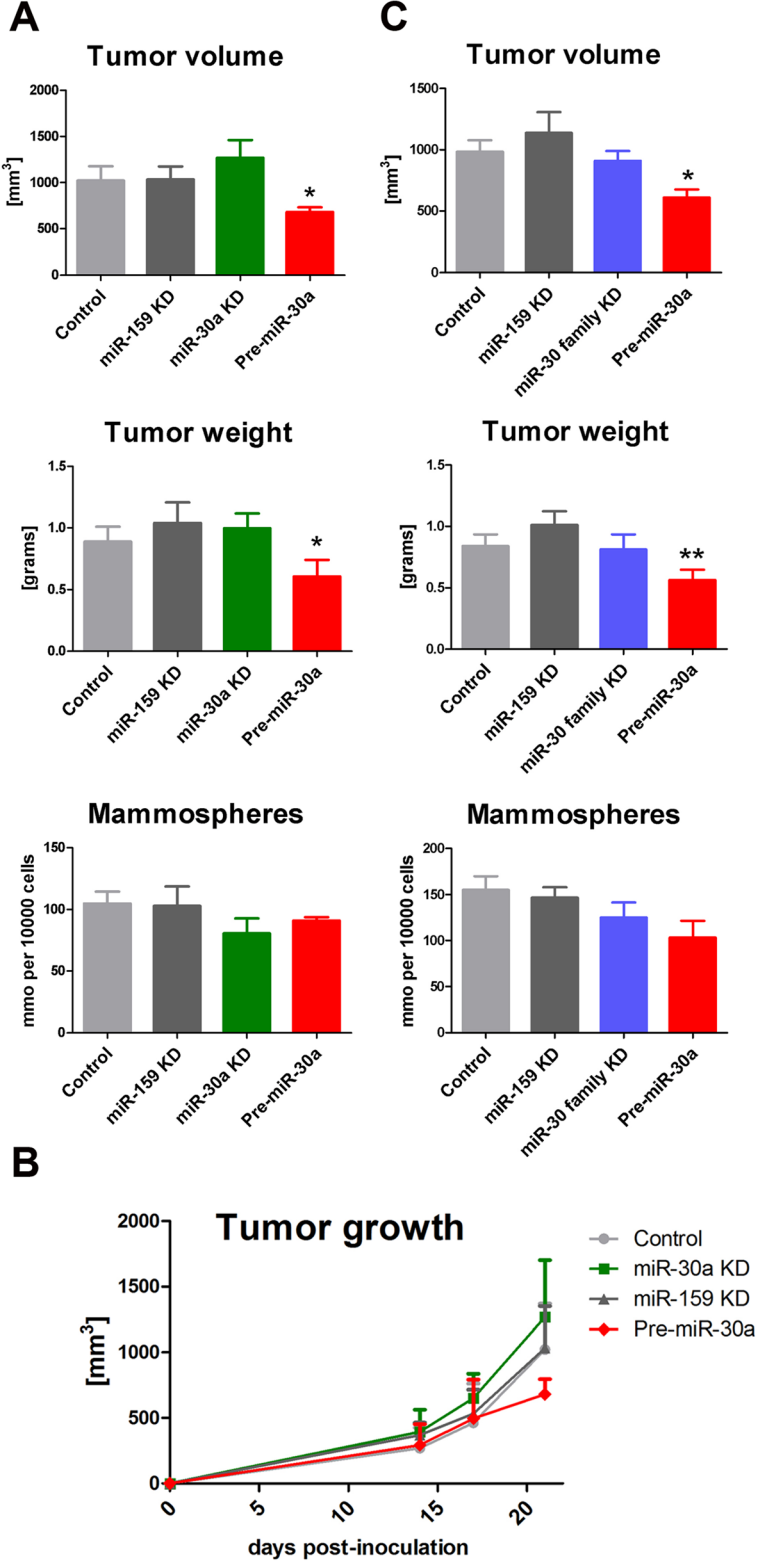
**Role of miR-30 in tumor progression and metastasis. A**. 4T1 mammary cancer cells were transiently transfected with miR-30a KD, Pre-miR-30a, and miR-159 KD control oligos. Next, cells were injected in the mammary fat pad of BALB/c mice (5–6 mice per group), and tumor volume (**left panel**) and weight (**central panel**) were registered after 3 weeks. At the end of the 3 weeks, tumors were disaggregated and plated in a mammosphere formation assay. Number of mammospheres was counted in triplicates for each condition (**right panel**). P value under 0.05 is represented with an asterisk (*). **B**. growth curve for the experiment described in (**A**). Quantification of tumor volume was performed from day 14 (when tumors are detectable) to day 21. **C**. An independent experiment was performed in a similar way to (**A**), using miR-30 family KD oligos instead of single miR-30a KD P value under 0.05 (two-tailed student t test) is represented with a (*).

To explore the in vivo role of miR-30a, we induced tumors by injecting 4T1 cells in the mammary fat pads of immunocompetent BALB/c mice. We chose 4T1 cells because its patterns of tumor growth at the primary site of injection and the metastatic spread in BALB/c mice closely mimic human breast cancer [19]. Prior to s.c. injection, 4T1 cells were transiently transfected with miR-30a-KD, pre-miR-30a or control miR-159-KD oligos. After 3 weeks, tumors induced with miR-30a overexpressing 4T1 cells (pre-miR-30) were significantly smaller and lighter than control and miR-30a KD tumors (Figure 5A and 5B). Tumours collected after 3 weeks were dissociated and plated under mammosphere conditions to quantify their sphere formation potential. Interestingly, cells derived from both, miR-30a-KD and pre-miR-30a, displayed a lower ability to form spheroids in vitro. Although this difference was not significant for miR-30a-KD, it was borderline significant for pre-miR-30a (p=0.07) (Figure 5A, lower panel). To further explore the potential role of other members of miR-30 family, this experiment was replicated using simultaneous inhibition of the miR-30 family (miR-30 family KD), as described above. Similar to the previous experiment, only miR-30a overexpression was able to induce significantly smaller tumors under these conditions (Figure 5B). No significant differences were observed after miR-30 family KD. In addition, a trend for a reduced number of ex-vivo spheroids was again observed with pre-miR-30a (Figure 5B, right panel). Importantly, several studies have suggested a metastatic behavior for putative BT-ICs. Therefore, we next studied the induction of lung metastasis under modulation of miR-30. A non-significant reduction in the number of metastatic colonies was observed after miR-30a overexpression (Additional file 7: Figure S5A and Figure S5B). Interestingly, while the number of mammospheres did not correlate significantly with tumor weight or volume, it significantly correlated with the number of colonies (r=0.37, pval=0.042). Therefore, a higher ability to produce spheres ex vivo correlated with a higher number of lung colonies in the clonogenic assay. This suggests a link between the ability to grow in non-attachment conditions and the metastatic behavior in vivo. Together, these in vivo experiments support an important role for miR-30 in regulating the mammary cancer cells responsible for tumor growth.

### AVEN overexpression can rescue mammosphere growth in presence of miR30a

**Figure 6:**
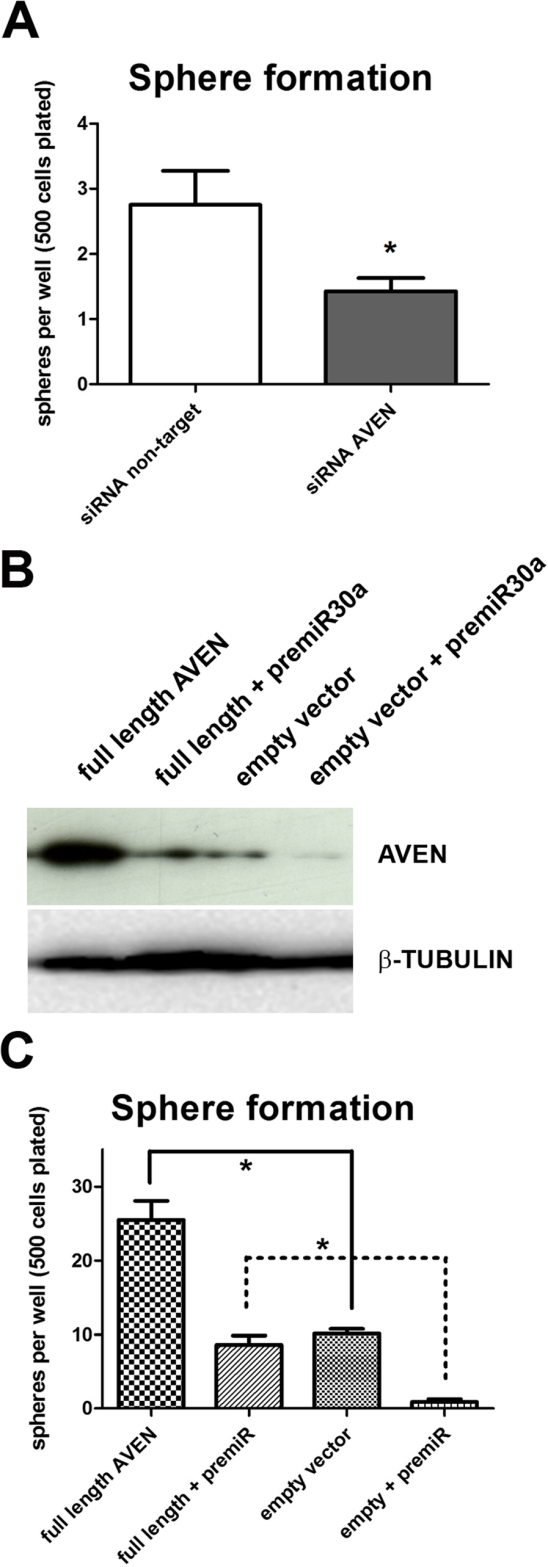
**Role of AVEN in non-attachment growth. A**. Sphere formation assays were performed 48 hours after transfection of MCF7 cells with esiRNA targeting AVEN. Sphere counting was performed as in Figure 2. **B**. MCF7 cells were transfected with empty pcDNA3.1 vector or a plasmid coding for full length AVEN, in combination or not with pre-miR30a oligos. Western blots were performed for the different conditions to test for efficiency of overexpression. Beta-tubulin was used as housekeeping control. **C**. The same conditions used in (**B**) were tested in a new sphere formation assay under non-attachment conditions. P values under 0.05 (two-tailed student t test) are represented with an asterisk (*). The sphere formation plots show the average and standard deviation of 8 independent non-attachment wells.

The biological role of miRNAs is the result of simultaneously targeting multiple transcripts [20]. In this sense, we expect that miR30 role in non-attachment growth is probably the result of multiple target regulation. However, we were interested in investigating in more detail the role of AVEN, because of the link between increased expression of this protein and cell survival [21], and poor prognosis in different types of human malignancies [22,23]. Moreover, the role of AVEN has been specifically addressed in breast cancer cells, including MCF7 cells [24]. We used endoribonuclease-prepared siRNA pools (esiRNA) targeting *AVEN* coding sequence to transiently silence *AVEN* expression in MCF7 cells. Two days after siRNA we tested the effect of *AVEN* silencing on a sphere formation assay, as described above. Importantly, we found that silencing significantly impaired sphere formation when compared to a non-targeting control siRNA (P<0.05) (Figure 6A). In addition, we used plasmid overexpression to further understand the role of AVEN in sphere formation. To this end, we used an expression plasmid encoding for full length *AVEN*. We selected a plasmid concentration that increased AVEN expression without affecting cell survival. Western blots performed 48 hours after transfection of MCF7 cells showed a higher AVEN expression relative to cells transfected with empty-vector (Figure 6B). Under non-attachment conditions, we found that increased expression of AVEN produced a significant increase in sphere formation, compared to empty pcDNA3.1 vector (Figure 6C). These results suggest that AVEN likely has a specific role in survival under non-attachment conditions in breast cancer cells. In addition, we assessed the ability of AVEN to rescue the negative effect of pre-miR30a transfection (miR-30a overexpression) in the sphere formation assay. Importantly, we observed lower expression of AVEN when overexpressing miR30a (pre-miR30a) in both conditions, empty vector and full length *AVEN* transfected cells. This is probably the result of miR30a targeting the endogenous *AVEN*, which is expressed at detectable levels under basal conditions (Figure 6B). Next, we validated the effect of miR30a overexpression in the presence of an empty pcDNA3.1 vector under non-attachment conditions (Figure 6C). miR30a overexpression drastically impairs the ability to form spheres. As described above, we observed a significant increase in the number of spheres after transfection with full length AVEN plasmid. Interestingly, full length *AVEN* was able to significantly increase sphere formation in the presence of miR30a overexpression to levels close to control empty-vector levels (Figure 6C).

The negative effect of *AVEN* silencing in sphere formation suggests an independent role of this protein in survival under non-attachment conditions. Moreover, the ability of *AVEN* to rescue miR30a effect suggests that the role of miR30a expression in non-attachment growth can be partially mediated through targeting of the transcript for this anti-apoptotic protein.

## Discussion

Due to their ability to simultaneously target multiple transcripts, miRNAs are able to participate in most cellular processes. In the same way, their deregulation has been frequently observed in complex human diseases, including cancer. In this report we studied the potential role of miRNAs in sustaining the subpopulation of breast cancer cells with the highest tumor-initiating ability. We identified miR-30 as a family of miRNAs strongly regulated under non-attachment conditions of cell growth, a standard method for selecting BT-ICs. By modulating the expression of miR-30 family we were able to regulate the growth in non-attachment conditions, as shown by sphere formation assays performed in vitro, and ex vivo. In addition, upregulation of miR-30 family expression impaired tumor growth in a mouse xenograft model. We were able to further identify potential common targets of miR-30 family with a role in survival and proliferation. These findings explain why a common downregulation of multiple members of the same miRNA family may be necessary for sustaining the growth in non-attachment conditions.

An important consideration is the absolute copy number of miRNAs necessary to target a specific mRNA. This has been recently addressed using high throughput assays (Sensor-seq) [25,26]. Interestingly, only a fraction of detected miRNAs (41%) displayed suppressive activity, and this activity correlated with a miRNA expression above 100 reads per million, or 100 copies per cell. Our absolute quantification of miR30a shows approximately 20 copies per MCF7 cell. Although this is significantly higher than the number of copies observed in mammospheres, the absolute copy number lies below those reported as biologically relevant [25,26]. This borderline number of copies may explain a relatively “mild” effect of miR30a knockdown. However, based on our own data, many miR30a targets were common targets to other members of the miR30 family. Therefore, the cumulative concentration of miR30 family may reach a level beyond biological suppressive activity. However, we were not able to see an increased effect on mammosphere formation after silencing the whole miR30 family (data not shown). Other considerations include the ratio between miRNA and target mRNA abundance, and the longer half life of miRNAs compared to mRNAs.

We performed the first comprehensive analyses of miR-30 family targets. Importantly, two recent studies combined proteomics and microarrays to reveal that changes in protein expression mediated by a miRNA are usually associated with altered mRNA expression, suggesting that mRNA degradation may be the major component of mammalian miRNA repression [4,5]. These recent findings give strong support to our strategy for target identification by using mRNA screening.

The importance of different miR-30 family members has been highlighted in several contexts. miR-30e was shown to regulate self-renewal and inhibit apoptosis in BT-ICs [27]. Overexpression of miR-30e in these cells inhibits their self-renewal capacity by reducing *Ubc9*, and induces apoptosis through silencing *ITGB3*. Although *Ubc9* and *ITGB3* were not included in our final list of miR-30a targets, we studied in more detail other targets also potentially involved in apoptosis and proliferation. FOXD1 (forkhead box protein 1) has a role in tumor formation [28], while AVEN (apoptosis, caspase activation inhibitor) has an established role in apoptosis regulation [21,24]. Although AVEN is unlikely to be the only miR-30a target involved in non-attachment growth, our results suggest an important role in this process that should be followed in further studies. For example, it would be interesting to understand the mechanisms by which AVEN rescues cell death, and whether they differ from the truncated deltaN-AVEN form [24]. In addition, because of the transient transfections used in our experiments, our results may be an underestimation of the role of miR30 and AVEN, and studies on stable/inducible systems may provide more drastic effects. In line with our findings, ectopic expression of miR-30 in BT-ICs xenografts reduced tumorigenesis and lung metastasis in non obese diabetic/severe combined immunodeficient mice, whereas blocking miR-30e expression enhanced tumorigenesis and metastasis [27]. In this sense, miR-30 downregulation may correlate with an in vitro expansion of putative BT-ICs. In addition, recent studies suggested a role of miR-30 family in epithelial-mesenchymal transition [29,30] and replicative senescence [31], processes closely linked to stem cell biology and tumor suppression, respectively.

A potential link between miR-30 expression and clinical parameters has also been shown. miR-30 was recently found to be part of a metastatic signature in a series of breast, bladder, colon and lung cancers [32]. Indeed miR-30c expression has been suggested as a predictor of endocrine therapy in ER+ breast cancer [33]. Interestingly, it was shown that mir-30 family members are all down-regulated in both estrogen receptor– and progesterone receptor–negative tumors, suggesting that expression of these miRNAs is regulated by these hormones [34]. Indeed, two members of the miR30 family have been recently shown to be downregulated by progestins [35]. In addition, miR-30a-5p, as well as miR-26a and miR-26b, were shown to be downregulated in tumors with high proliferation index [34].

## Conclusion

Our study indicates that putative BT-ICs enriched in a mammosphere assay have a distinct miRNA profile, essential for their proliferation balance. In vitro, this distinct profile is necessary to acquire the capacity to grow in non-attachment conditions. In vivo, this profile may be involved in a higher ability to induce tumors. We highlight the specific role of miR-30 family in these two contexts, and performed the first comprehensive analyses of miR-30 family targets.

## Methods

### Ethics statement

The animal studies have been approved by the Animal Care Committee of University of Ottawa. All mice received normal diet and were monitored daily by the Animal Care and Veterinary Service-(ACVS) staff. Mice did not receive any invasive treatment except one time subcutaneous (s.c.) injection of 4T1 cancer cells. The experimental endpoint was a total sacrifice 3 weeks after cancer cells inoculation. It was chosen to prevent physiological changes (walking and moving) of mice due to tumor size and to avoid tumor necrosis. Method of euthanasia: Mice received injectable Ketamine/Xylazine before cervical dislocation (0.1 ml of a mix of Ketamine [200 mg/kg] and Xylazine [100 mg/kg] via IP). The standards for animal care and use conform with or exceed those defined in the Canadian Council on Animal Care’s Guide to the Care and Use of Experimental Animals, Vol. 1, 2nd edn., 1993 and the Animals for Research Act, R.S.O. 1990, c. A.22, s. 17. Study protocol number ME-259.

### Cell culture and mammosphere production

Breast cancer cell lines (American Type Culture Collection) were grown in standard (10% fetal calf serum, 1% penicillin/streptomycin, 1% sodium pyruvate and 1% glutamine) medium. Slightly attached cells from semi-confluent culture dishes were centrifuged and plated in mammosphere conditions, as previously described [36]. Alternatively, cells were counted and tested for viability with Trypan blue after trypsinization, and plated under non-attachment conditions with mammosphere medium.

### Transfections and luciferase assays

Pre-miR mature microRNA (Pre-miR-30a) sequence (Applied Biosystems) and knock-down (miR-30a-KD) locked nucleic acid (LNA) (Exiqon) were used for overexpression and inhibition of miR-30a, respectively. miR-159 LNA was used as negative control. Inhibition of the five members of the miR-30 family (a,b,c,d,e) was obtained using the equimolar mix of two oligos, oligo 1 targeting the miR-30a, miR-30d and miR-30e, and oligo 2 targeting miR-30b and miR-30c (Exiqon, custom miRNA family knock-down design).

For luciferase assays, MCF7 cells plated in 24-well plates were transfected with 0.8 μg of the empty pGL3-Basic, pGL3-Control, pGL3-FOXD1-3^′^UTR, or pGL3-FOXD1/mut-3^′^UTR. Assays were performed 48h after transfection using the Dual Luciferasse Reporter Assay system (Promega), and normalized with Renilla luciferase activity. All transfections were performed using Lipofectamine 2000 (Invitrogen) according to the manufacturer’s instructions.

Plasmid for AVEN expression assays (full length and control) ligated into a pcDNA3.1 vector, were a kind gift from I. Melzer (Frankfurt, Germany), and have been previously described [24]. For silencing of *AVEN* we used commercially available endoribonuclease-prepared siRNA pools (esiRNA) targeting *AVEN* coding sequence (Sigma).

### Transcriptome and MicroRNA expression arrays

Total RNA was extracted using TRIzol (Sigma) according to the manufacturer’s instructions. For miRNA expression, RNA labeling and hybridization on Ohio State University miRNA microarray chips (OSU CCC rel. 4.0) were done as described elsewhere [37]. For transcriptome analyses, total RNA from cells transfected with Pre-miR-30, KD-miR-30a, KD-miR-30 family or control KD-miR-159 was reverse-transcribed (Ambion Illumina Total Prep) and hybridized on HT12 Human bead chips (Illumina). Validation of miRNA microarray was performed on Illumina Human v2 microarray, containing 1146 miRNA probes (covering 95% of miRBase1 v12.0 known human miRNAs). Both, miRNA and mRNA expression were also validated using TaqMan (Applied Biosystems) and SyBR green (Eurogentec) quantitative RT-PCR, respectively. Microarray raw data has been deposited in the Gene Expression Omnibus repository under the accession number GSE36565 (submitter: Hernandez-Vargas H).

### Mouse model experiments

4T1 cells were cultured in RPMI-1640 media (ATCC) containing 10% FBS, penicillin/streptomycin (0.05 mg/mL) at 37°C in a humidified atmosphere with 5% CO2. Six- to 8-week-old BALB/c female mice weighting 18–20 g (Charles River, Montreal, QC) were randomly distributed into experimental groups: control, miRNA-30a-KD, miRNA-159-KD, miRNA-30-family KD, and pre-miR-30a (5–6 mice per group). Mice were housed in a controlled atmosphere (temperature 22 ± 2°C; humidity 55 ± 2%) with a 12 h light/dark cycle. Mice were injected s.c. with transfected 4T1 cells (1400 cells/0.1 ml/ mice) into the second left mammary gland fat pad. After three weeks, tumors were collected and weighted. Approximately 0.05 g of each tumor was minced and dissociated in RPMI-1640 media containing 300 U/ml collagenase (Sigma), and 100 U/ml hyaluronidase (Sigma) at 37°C for 2 h. Cells were sieved sequentially through a 100 μm and a 40 μm cell strainer (BD Biosciences) to obtain a single cell suspension, and counted in a haemocytometer. Single cells were plated in ultralow attachment 96-well plates (Costar) at 104 cells/0.2 ml/well in DMEM-F12 (#12660, Invitrogen), supplemented with 10 ng/ml EGF, 20 ng/ml bFGF, 5 μg/ml insulin, 1 mM sodium pyruvate, 0.5 μg/ml hydrocortisone, and penicillin/streptomycin (0.05 mg/mL). Cells grown in these conditions as nonadherent spherical clusters of cells (mammospheres) were counted after 7 days.

4T1 cells were also used to study metastatic behavior. Because 4T1cells are resistant to 6-thioguanine, micro-metastatic cells (as few as 1) can be detected in many distant site organs with better accuracy that most tumour models. Lungs obtained after the different conditions were minced and dissociated in RPMI-1640 media containing 300 U/ml collagenase (#C7657, Sigma), at 4°C for 75 min. After the filtration through a 40 μm cell strainer (BD Biosciences), cells were collected and resuspended in RPMI-1640 containing 10% FBS (ATCC), penicillin/streptomycin (0.05 mg/mL) and 60 μM 6-thioguanine 60 (Sigma). Cells were plated in 10-cm culture dishes (Corning) at 37°C in a humidified atmosphere with 5% CO2. After 14 days, cells were fixed by methanol and stained with 0.03% methylene blue solution. All blue colonies were counted, one colony representing one clonogenic metastatic cell.

### Statistical analyses

BRBArrayTools v3.8.1 was used for bead array analysis, as described above. For other comparisons, means and differences of the means with 95% confidence intervals were obtained using GraphPad Prism (GraphPad Software Inc.). Two-tailed student t test was used for unpaired analysis comparing average expression between classes. Pearson’s correlation was used to study the association between mammosphere formation and metastatic behavior. P values < 0.05 were considered statistically significant.

Raw miRNA and transcriptome data were background subtracted, quantile normalized, and further analyzed by BRB-Array Tools Version 3.8.1 (developed by Dr. Richard Simon and the BRB-ArrayTools Development Team). For quantile normalization we used the median array as the reference array. The normalization is performed by computing a gene-by-gene difference between each array and the reference array, and subtracting the median difference from the log-intensities on that array, so that the gene-by-gene difference between the normalized array and the reference array is 0.

Gene ontology analyses were performed with the Database for Annotation, Visualization and Integrated Discovery (DAVID) v6.7 using the whole human genome as reference. In addition, geneset enrichment was done in BRB-Array Tools for Gene Ontology categories, and biological pathways (BioCarta and KEGG).

For class comparison analyses p value < 0.001 and false discovery rate (FDR) < 0.15 were used as cut-offs. Genes significantly distinguishing the classes were further analyzed with the miRecords resource (http://mirecords.biolead.org/prediction_query.php) to identify predicted targets for miR-30. Only genes predicted by at least 6 out of 10 miRNA predicting tools were taken into account. PITA algorithm (http://genie.weizmann.ac.il/pubs/mir07/mir07_dyn_data.html) was used to identify the seed sequences for each gene.

## Abbreviations

AVEN: Apoptosis, caspase activation inhibitor; BT-ICs: Breast tumor initiating cells; FOXD1: Forkhead box protein 1; FDR: False discovery rate; KD: Knock-down; miR: microRNA; qRT-PCR: Quantitative reverse transcription-polymerase chain reaction; s.c.: Subcutaneous; UTR: Unstranslated region

## Competing interests

The authors disclose no potential conflicts of interest.

## Authors’ contributions

M.O., T.V., P.A., and M.F performed all the experiments. H.H., C.M. and Z.H. coordinated the project. C.C. coordinated the microRNA oligonucleotide array experiment. F.L. and G.D. coordinated and performed the Illumina expression bead arrays. M.O. and H.H. performed the bioinformatics analyses and wrote the manuscript. All authors discussed the results and manuscript text. All authors read and approved the final manuscript.

## Supplementary Material

Additional file 1: Figure S1 miRNA profiling in MCF7 cells, MCF7-derived mammospheres (MMO), and differentiated mammospheres (DIFF MMO). miRNA profiling was performed using an oligonucleotide array. **A.** Unsupervised cluster analysis and heat-map including all probes. Clustering of all mammosphere samples indicate a defined profile, distinct from MCF7 and DIFF MMO. **B.** Volcano plots of fold change (represented in Log2 ratio in the x axis) vs. P value (represented in Log10 ratio in the y axis). Volcano plots indicate a subset of miRNAs downregulated in MMO, respective to MCF7 and DIFF MMO. For comparison, MCF7 and DIFF MMO (in the lower panel) display a very similar profile. Click here for file

Additional file 2 **Table S1.** miRNAs differentially expressed among MCF7 cells, MCF7-derived mammospheres, and differentiated mammospheres. **Table S2** putative targets of miR-30 after WGE analysis of miR-30 family KD and Pre-miR-30a. Click here for file

Additional file 4: Figure S3 Absolute quantification of miR-30a copy number. **A**. Standards of synthetic miR30a oligonucleotide were used at known nanomolar concentration in a TaqMan qRT-PCR assay. Amplification plots are shown in the left panel, and standard curve is shown in the right panel, with corresponding efficiency and correlation values. Biological samples were run simultaneously with known miR30a standards in TaqMan qRT-PCRs. After accounting for the different dilutions and original number of cells, absolute number of copies per cell was obtained for parental MCF7 cells compared to MCF7-derived mammospheres (**B**), and MCF7 cells treated with different experimental conditions (MCF7 controls, miR30a overexpression and knock-down) (**C**). P values under 0.05 (two-tailed student t test) are represented with an asterisk (*). Click here for file

Additional file 3: Figure S2 Validation of miRNA expression using Illumina platform. miRNA profiling was validated using Illumina bead arrays in independent biological replicates of MMO and parental MCF7 cells. **A**. Scatter plot between 2 technical replicates indicating a proper correlation. **B**. Unsupervised cluster analysis and heat-map including all probes. **C**. Volcano plots of fold change (represented in Log2 ratio in the x axis) vs. P value (represented in Log10 ratio in the y axis). Volcano plots indicate a subset of miRNAs downregulated in MMO, respective to MCF7 cells. **D**. Expression of selected miRNAs significantly downregulated in MMO, relative to parental MCF7 cells. Click here for file

Additional file 5: Figure S4 Whole genome expression after modulation of miR-30a expression. MCF7 cells were transiently transfected with different conditions, miR-30a-k.d., Pre-miR-30a, and control miR-159-k.d. After 48 hours, RNA was extracted from biological triplicates, and interrogated for whole genome expression with Illumina HT12 bead arrays. **A**. Scatter plot between 2 technical replicates indicating a proper correlation. **B**. Cluster analysis and heat-map including all probes differentially expressed between Pre-miR-30a and miR-159 control k.d. As can be seen, no transcripts were significantly different between miR-30a-k.d. and miR-159 control k.d. **C**. Expression of selected miRNAs was validated by qRT-PCR in independent biological triplicates. Downregulation was as expected in cells overexpressing miR-30a (Pre-miR-30a), while no differences were found after miR-30a k.d, confirming the results from the whole genome expression assay. P values under 0.05 (two-tailed student t test) are represented with an asterisk (*). Click here for file

Additional file 6 ** 420 gene sets sorted by LS permutation p-value (significant p-values are in red).** Table of Gene Sets: 42 gene sets sorted by LS permutation p-value (significant p-values are in red). Table of Gene Sets: 43 gene sets sorted by LS permutation p-value (significant p-values are in red). Click here for file

Additional file 7: Figure S5 Lung metastasis assay after modulation of miR30 expression. 4T1 mammary cancer cells were transiently transfected with miR-30a family KD, Pre-miR-30a, and miR-159 KD control oligos. Next, cells were injected in the mammary fat pad of BALB/c mice and tumor growth was followed during 21 days (experiment shown in Figure 5C). Lungs obtained after the different conditions were minced, dissociated, and cells were suspended in medium containing 6-thioguanine and plated in culture dishes. After 14 days, cells were fixed by methanol and stained with methylene blue solution (**A**). All blue colonies were counted, one colony representing one clonogenic metastatic cell (**B**). Click here for file
