# Supplementary figures and images for: MicroRNA miR-30 family regulates non-attachment growth of breast cancer cells
Source: BMC Genomics. 2013 Feb 28;14:139. doi: 10.1186/1471-2164-14-139 (PMC3602027; doi:10.1186/1471-2164-14-139)

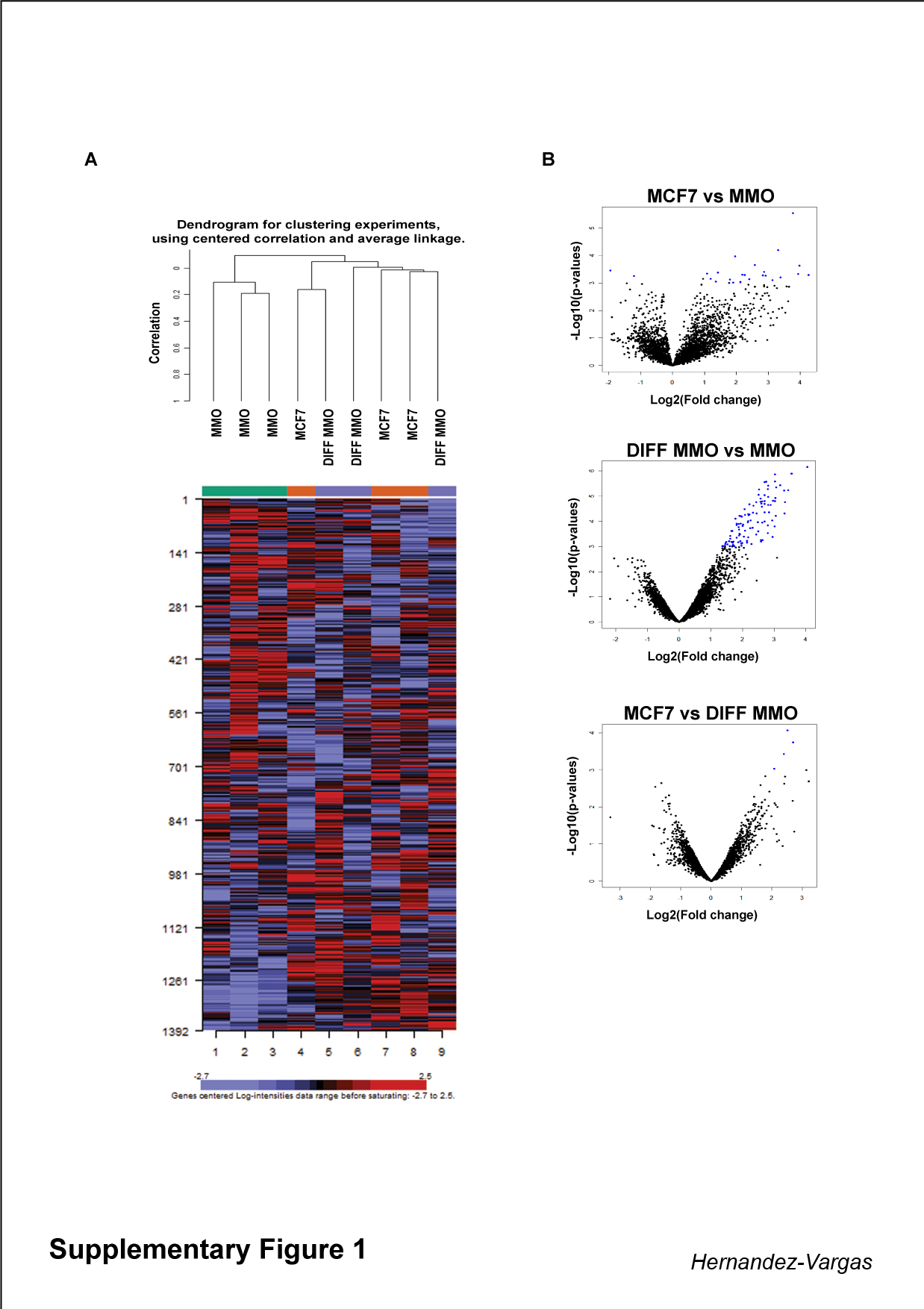

Supplement: Additional file 1: Figure S1 — miRNA profiling in MCF7 cells, MCF7-derived mammospheres (MMO), and differentiated mammospheres (DIFF MMO). miRNA profiling was performed using an oligonucleotide array. A. Unsupervised cluster analysis and heat-map including all probes. Clustering of all mammosphere samples indicate a defined profile, distinct from MCF7 and DIFF MMO. B. Volcano plots of fold change (represented in Log2 ratio in the x axis) vs. P value (represented in Log10 ratio in the y axis). Volcano plots indicate a subset of miRNAs downregulated in MMO, respective to MCF7 and DIFF MMO. For comparison, MCF7 and DIFF MMO (in the lower panel) display a very similar profile. [file 1471-2164-14-139-S1.tiff]

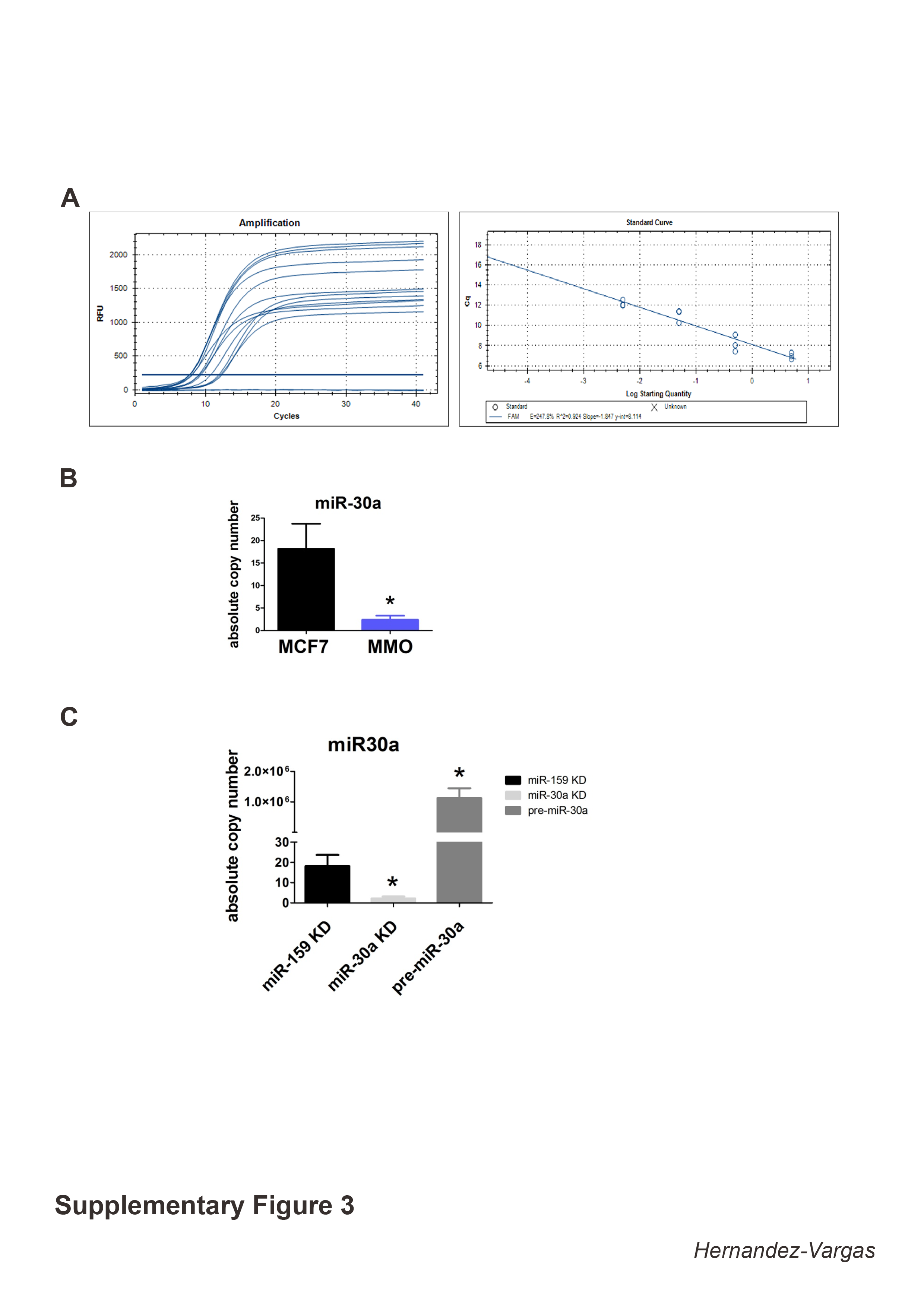

Supplement: Additional file 4: Figure S3 — Absolute quantification of miR-30a copy number. A. Standards of synthetic miR30a oligonucleotide were used at known nanomolar concentration in a TaqMan qRT-PCR assay. Amplification plots are shown in the left panel, and standard curve is shown in the right panel, with corresponding efficiency and correlation values. Biological samples were run simultaneously with known miR30a standards in TaqMan qRT-PCRs. After accounting for the different dilutions and original number of cells, absolute number of copies per cell was obtained for parental MCF7 cells compared to MCF7-derived mammospheres (B), and MCF7 cells treated with different experimental conditions (MCF7 controls, miR30a overexpression and knock-down) (C). P values under 0.05 (two-tailed student t test) are represented with an asterisk (*). [file 1471-2164-14-139-S4.tiff]

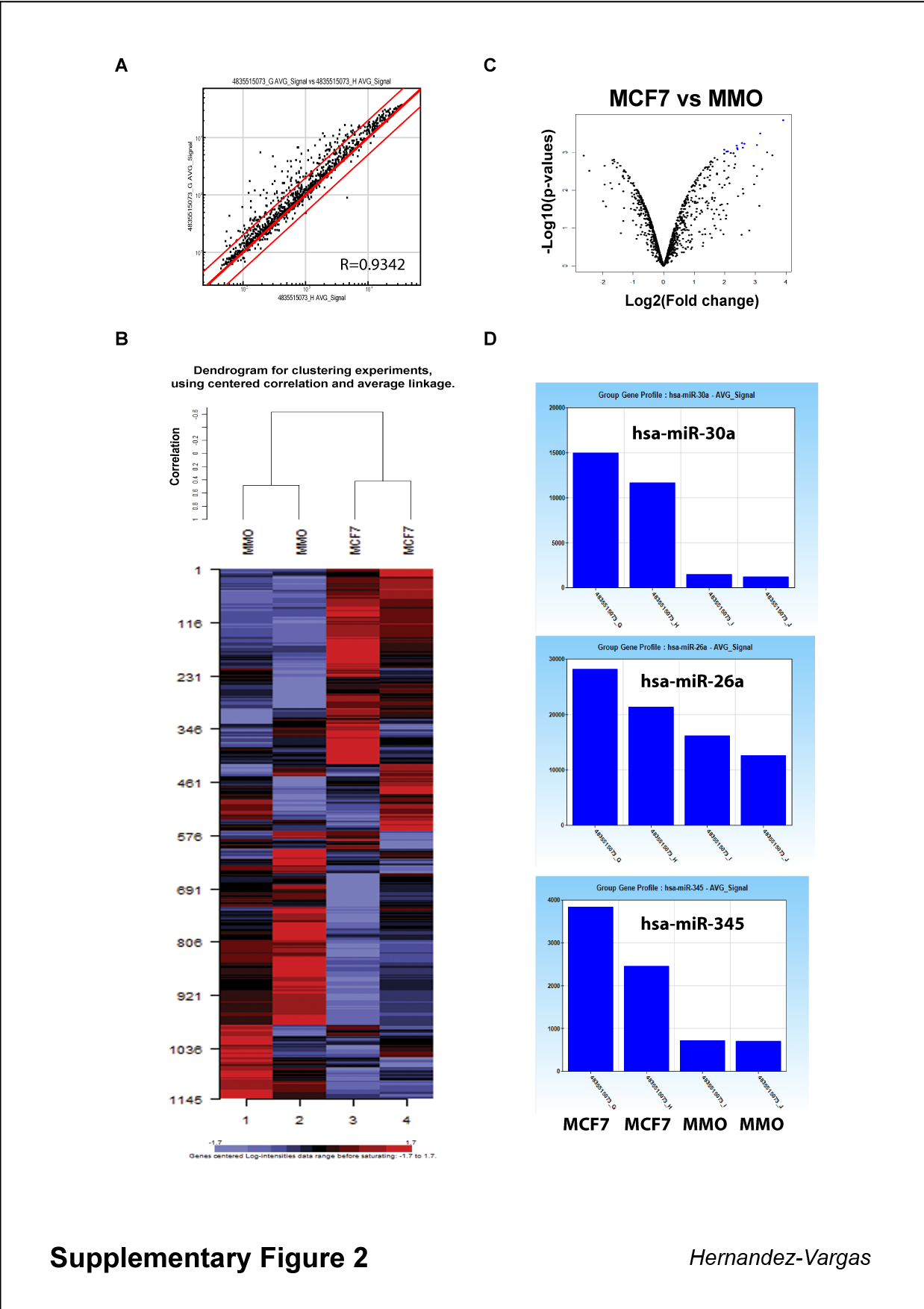

Supplement: Additional file 3: Figure S2 — Validation of miRNA expression using Illumina platform. miRNA profiling was validated using Illumina bead arrays in independent biological replicates of MMO and parental MCF7 cells. A. Scatter plot between 2 technical replicates indicating a proper correlation. B. Unsupervised cluster analysis and heat-map including all probes. C. Volcano plots of fold change (represented in Log2 ratio in the x axis) vs. P value (represented in Log10 ratio in the y axis). Volcano plots indicate a subset of miRNAs downregulated in MMO, respective to MCF7 cells. D. Expression of selected miRNAs significantly downregulated in MMO, relative to parental MCF7 cells. [file 1471-2164-14-139-S3.tiff]

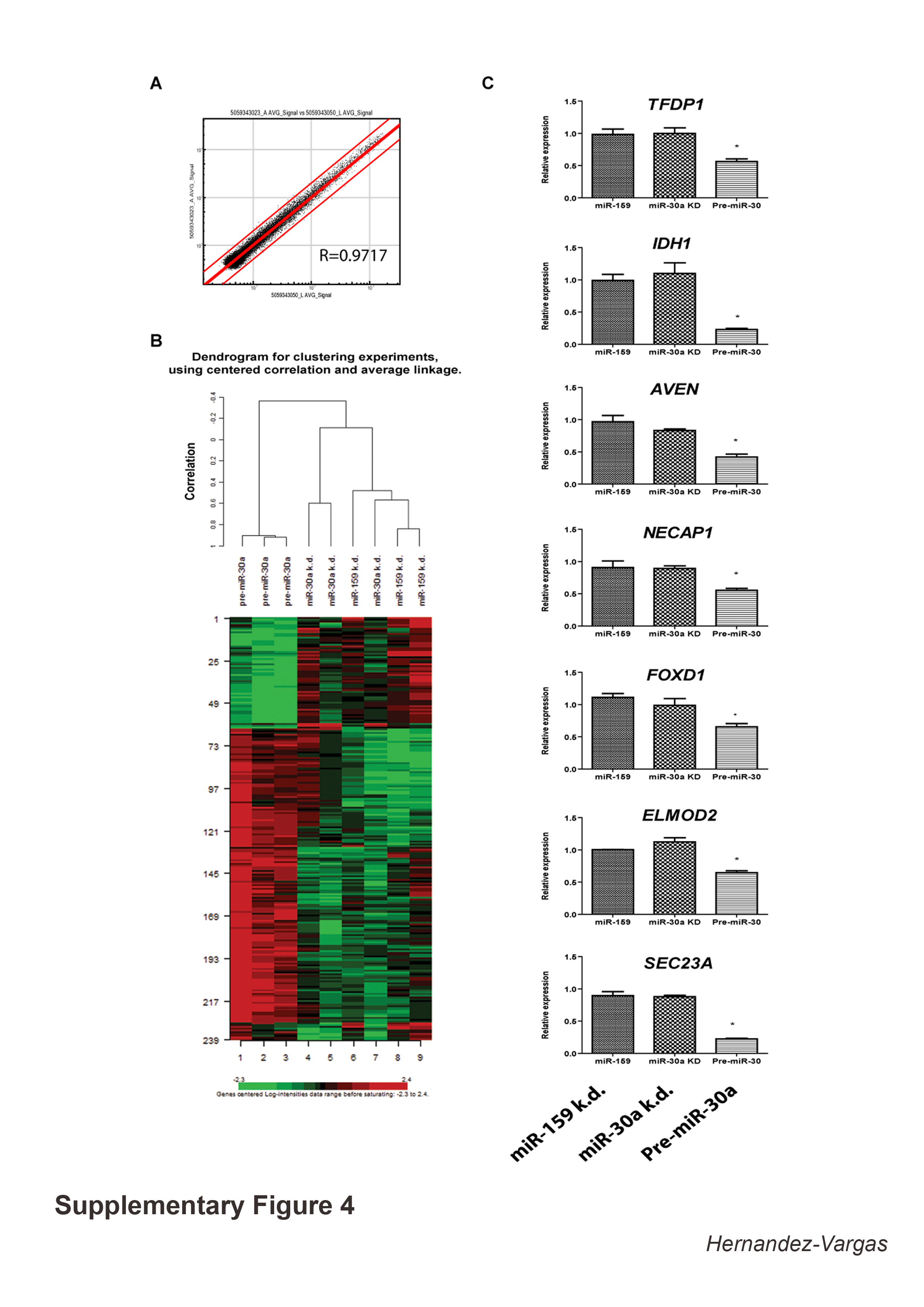

Supplement: Additional file 5: Figure S4 — Whole genome expression after modulation of miR-30a expression. MCF7 cells were transiently transfected with different conditions, miR-30a-k.d., Pre-miR-30a, and control miR-159-k.d. After 48 hours, RNA was extracted from biological triplicates, and interrogated for whole genome expression with Illumina HT12 bead arrays. A. Scatter plot between 2 technical replicates indicating a proper correlation. B. Cluster analysis and heat-map including all probes differentially expressed between Pre-miR-30a and miR-159 control k.d. As can be seen, no transcripts were significantly different between miR-30a-k.d. and miR-159 control k.d. C. Expression of selected miRNAs was validated by qRT-PCR in independent biological triplicates. Downregulation was as expected in cells overexpressing miR-30a (Pre-miR-30a), while no differences were found after miR-30a k.d, confirming the results from the whole genome expression assay. P values under 0.05 (two-tailed student t test) are represented with an asterisk (*). [file 1471-2164-14-139-S5.tiff]

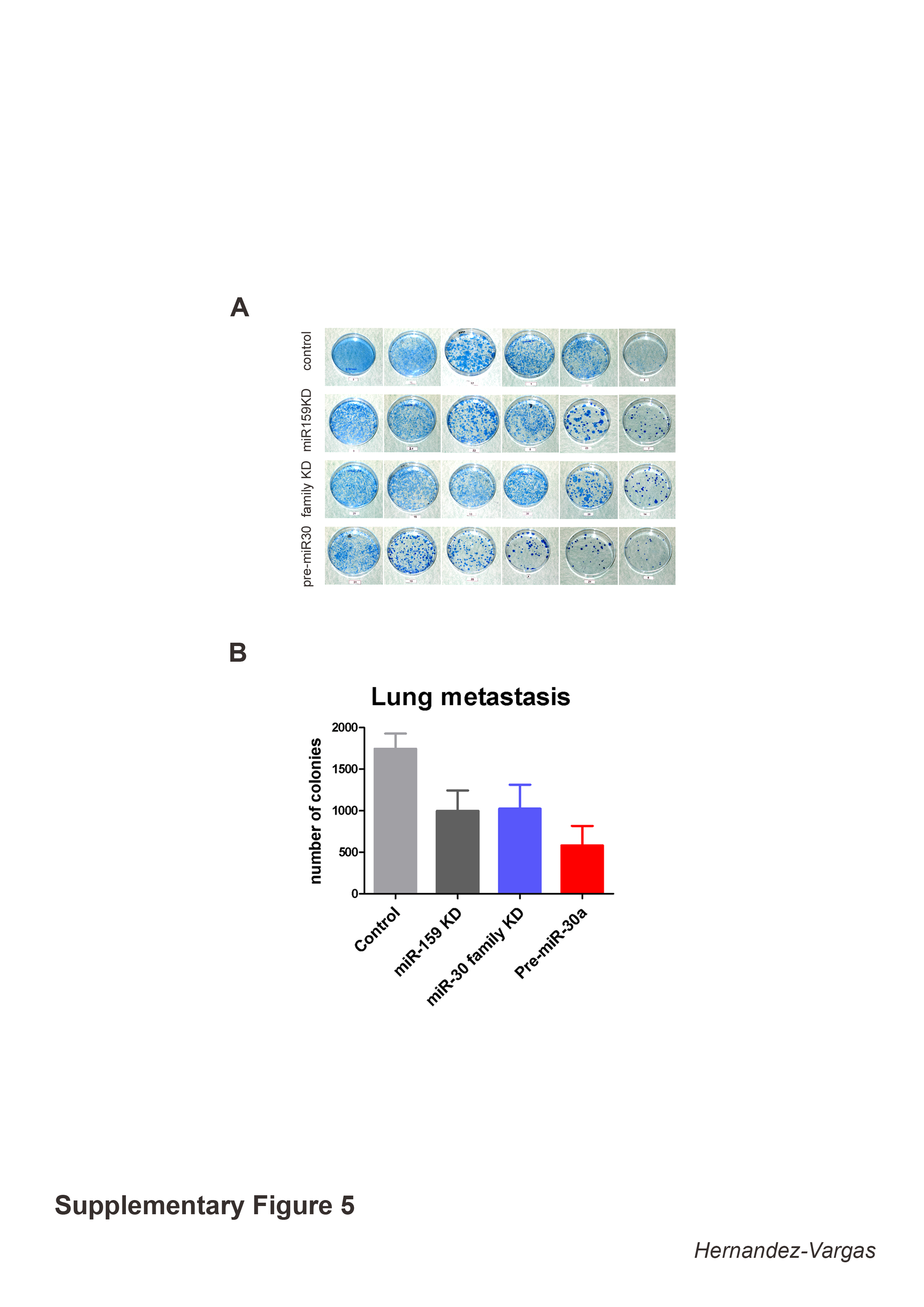

Supplement: Additional file 7: Figure S5 — Lung metastasis assay after modulation of miR30 expression. 4T1 mammary cancer cells were transiently transfected with miR-30a family KD, Pre-miR-30a, and miR-159 KD control oligos. Next, cells were injected in the mammary fat pad of BALB/c mice and tumor growth was followed during 21 days (experiment shown in Figure 5C). Lungs obtained after the different conditions were minced, dissociated, and cells were suspended in medium containing 6-thioguanine and plated in culture dishes. After 14 days, cells were fixed by methanol and stained with methylene blue solution (A). All blue colonies were counted, one colony representing one clonogenic metastatic cell (B). [file 1471-2164-14-139-S7.tiff]
