# Supplementary material for: MicroRNA miR-30 family regulates non-attachment growth of breast cancer cells
Source: BMC Genomics. 2013 Feb 28;14:139. doi: 10.1186/1471-2164-14-139 (PMC3602027; doi:10.1186/1471-2164-14-139)
Supplement: Additional file 2 — Table S1. miRNAs differentially expressed among MCF7 cells, MCF7-derived mammospheres, and differentiated mammospheres. Table S2 putative targets of miR-30 after WGE analysis of miR-30 family KD and Pre-miR-30a. [file 1471-2164-14-139-S2.doc]

**MicroRNA miR-30 family regulates non-attachment growth of breast cancer cells**

Maria Ouzounova^1^, Tri Vuong^2^, Mylène Ferrand^1^, Geoffroy Durand^3^, Florence Le-Calvez Kelm^3^, Carlo Croce^4^, Chantal Matar^2^, Zdenko Herceg^1^, and Hector Hernandez-Vargas^1^

*1. Epigenetics Group. International Agency for Research on Cancer (IARC). 150 rue Albert-Thomas, 69008 Lyon, France.*

*2. Faculty of Health Sciences, University of Ottawa. Ottawa, Canada K1H 8M5.*

*3. Genetic Cancer Susceptibility Group. International Agency for Research on Cancer (IARC). 150 rue Albert-Thomas, 69008 Lyon, France.*

*4. Ohio State University. 1082 Biomedical Research Tower, 460 W 12th Ave. Columbus, OH 43210, USA.*

**Supplementary Tables**

***Supplementary Table 1. miRNAs differentially expressed among MCF7 cells, MCF7-derived mammospheres, and differentiated mammospheres. Microarray data was filtered for those miRNAs displaying higher variability (more than 2-fold change in expression in at least 30% of samples). Significance thresholds: p value < 0.001, FDR < 0.1. Group codes for pairwise significance: group 1: DIFF MMO, group 2: MCF7, group 3: MMO.***

|  |  |  |  |  |  |  |  |
| --- | --- | --- | --- | --- | --- | --- | --- |
|  | **p-value** | **FDR** | **DIFF MMO** | **MCF7** | **MMO** | **Symbol** | **Pairwise significant** |
|  |  |  |  |  |  |  |  |
| **1** | 5.3E-05 | 3.8E-02 | 240.28 | 355.66 | 43.7 | **hsa-mir-30a-5p-A** | (3, 1), (3, 2) |
| **2** | 5.4E-05 | 3.8E-02 | 463.39 | 670.54 | 59.69 | **hsa-mir-30c-A** | (3, 1), (3, 2) |
| **3** | 1.3E-04 | 5.0E-02 | 383.16 | 566.72 | 44.93 | **hsa-mir-30b-A** | (3, 1), (3, 2) |
| **6** | 2.3E-04 | 5.2E-02 | 154.66 | 177.2 | 41.28 | **hsa-mir-130b-A** | (3, 1), (3, 2) |
| **7** | 3.4E-04 | 6.8E-02 | 21.72 | 6.16 | 1.44 | **hsa-mir-16-2-P** | (2, 1), (3, 1), (3, 2) |
| **10** | 5.0E-04 | 7.0E-02 | 978.78 | 1393.46 | 142.04 | **hsa-mir-30d-A** | (3, 1), (3, 2) |
| **13** | 7.4E-04 | 7.8E-02 | 281.94 | 289.3 | 13.11 | **hsa-mir-30c-1-A** | (3, 1), (3, 2) |
| **16** | 9.9E-04 | 7.9E-02 | 174.01 | 189.22 | 43.32 | **hsa-mir-181c-A** | (3, 1), (3, 2) |
| **17** | 1.0E-03 | 7.9E-02 | 193.16 | 201.24 | 17.16 | **hsa-mir-26a-2-A** | (3, 1), (3, 2) |
| **24** | 1.8E-03 | 1.0E-01 | 3539.69 | 2890.21 | 470.48 | **hsa-mir-23a-A** | (3, 1), (3, 2) |
| **29** | 2.7E-03 | 1.3E-01 | 272.85 | 393.82 | 77.5 | **hsa-mir-24-3p-A** | (3, 1), (3, 2) |
| **38** | 5.6E-03 | 1.9E-01 | 809.71 | 1060.61 | 178.84 | **hsa-mir-103-2-A** | (3, 1), (3, 2) |
| **40** | 5.8E-03 | 1.9E-01 | 95.97 | 178.09 | 22.34 | **hsa-mir-345-A** | (3, 2) |
| **44** | 6.4E-03 | 2.0E-01 | 6.93 | 5.72 | 21.08 | **hsa-mir-186-A** | (1, 3), (2, 3) |
| **48** | 7.5E-03 | 2.2E-01 | 136.75 | 196.43 | 36.18 | **hsa-mir-625-P** | (3, 2) |
| **58** | 1.0E-02 | 2.4E-01 | 263.65 | 293.52 | 29.17 | **hsa-mir-26a-A** | (3, 1), (3, 2) |
| **62** | 1.2E-02 | 2.7E-01 | 8.43 | 1.42 | 2.12 | **hsa-mir-325-P** | (2, 1) |
| **65** | 1.3E-02 | 2.8E-01 | 289.42 | 316.14 | 70.79 | **hsa-mir-181a1-5p-A** | (3, 2) |
| **71** | 1.4E-02 | 2.8E-01 | 667.08 | 711.56 | 126.92 | **hsa-mir-103-1-A** | (3, 2) |
| **72** | 1.6E-02 | 3.1E-01 | 9.12 | 2.05 | 9.55 | **hsa-mir-520b-A** | (2, 3) |
| **80** | 2.0E-02 | 3.4E-01 | 13.78 | 2.33 | 7.95 | **hsa-mir-526a-1-P** | (2, 1) |
| **85** | 2.1E-02 | 3.4E-01 | 26.84 | 45.6 | 2.13 | **hsa-mir-367-A** | (3, 2) |
| **97** | 2.8E-02 | 4.0E-01 | 272.51 | 293.88 | 42.46 | **hsa-mir-26a-1-A** |  |
| **96** | 2.8E-02 | 4.0E-01 | 7.88 | 38.46 | 67.01 | **hsa-mir-647-P** |  |
| **95** | 2.8E-02 | 4.0E-01 | 418.26 | 268.22 | 95.65 | **hsa-mir-801-P** |  |
| **102** | 2.9E-02 | 4.0E-01 | 5.03 | 5.04 | 29.24 | **hsa-mir-514-2&3-A** |  |
| **107** | 3.1E-02 | 4.0E-01 | 37.86 | 18.94 | 9.38 | **hsa-mir-562-A** |  |
| **108** | 3.1E-02 | 4.1E-01 | 15.15 | 5.76 | 30.62 | **hsa-mir-518a1-5p/mir527-A** | |
| **111** | 3.3E-02 | 4.1E-01 | 13 | 8.18 | 36.76 | **hsa-mir-144-A** |  |
| **113** | 3.4E-02 | 4.2E-01 | 1428.66 | 1558.29 | 449.71 | **hsa-mir-99b-A** |  |
| **118** | 3.8E-02 | 4.5E-01 | 7.12 | 3.78 | 16.92 | **hsa-mir-573-P** |  |
| **122** | 4.0E-02 | 4.5E-01 | 2.79 | 6.6 | 13.38 | **hsa-mir-517*a-5p-A** |  |
| **124** | 4.0E-02 | 4.5E-01 | 9.27 | 15.24 | 29.15 | **hsa-mir-520g-P** |  |
| **129** | 4.5E-02 | 4.8E-01 | 2.92 | 18.16 | 9.94 | **hsa-mir-513-1-P** |  |
| **128** | 4.5E-02 | 4.8E-01 | 4.32 | 9.68 | 23.16 | **hsa-mir-638-P** |  |
| **135** | 4.9E-02 | 5.1E-01 | 37.72 | 28.86 | 7.25 | **hsa-mir-105-2-A** |  |
| **143** | 5.4E-02 | 5.2E-01 | 377.74 | 311.38 | 101.86 | **hsa-mir-565-P** |  |
| **148** | 5.6E-02 | 5.3E-01 | 7.96 | 14.95 | 36.19 | **hsa-mir-506-A** |  |
| **145** | 5.6E-02 | 5.3E-01 | 37.08 | 42.99 | 9.93 | **hsa-miR-769-5p-A** |  |
| **174** | 6.7E-02 | 5.3E-01 | 60.91 | 86.76 | 16.93 | **hsa-mir-21No1** |  |
| **173** | 6.7E-02 | 5.3E-01 | 189.86 | 249.98 | 116.49 | **hsa-mir-302a*-5p-A** |  |
| **177** | 6.8E-02 | 5.3E-01 | 0.8 | 4.28 | 2.46 | **hsa-mir-34c-A** |  |
| **158** | 6.4E-02 | 5.3E-01 | 12.89 | 5.91 | 42.35 | **hsa-mir-487a-P** |  |
| **157** | 6.3E-02 | 5.3E-01 | 9.24 | 11.46 | 31.12 | **hsa-mir-525*-3p-A** |  |
| **183** | 7.3E-02 | 5.5E-01 | 71.96 | 47.47 | 16.39 | **hsa-mir-563-P** |  |
| **188** | 7.9E-02 | 5.8E-01 | 107.16 | 74.1 | 29.4 | **hsa-mir-193b-A** |  |
| **187** | 7.8E-02 | 5.8E-01 | 1465.33 | 1150.34 | 473.46 | **hsa-mir-375-A** |  |
| **195** | 8.1E-02 | 5.8E-01 | 8.92 | 31.71 | 10.33 | **hsa-miR-542-5p-A** |  |
| **197** | 8.2E-02 | 5.8E-01 | 7.02 | 4.57 | 20.86 | **hsa-mir-588-A** |  |
| **193** | 8.0E-02 | 5.8E-01 | 224.14 | 52.29 | 43.33 | **hsa-mir-801-A** |  |
| **201** | 8.4E-02 | 5.8E-01 | 3.97 | 11.19 | 17.07 | **hsa-mir-514-1-P** |  |
| **208** | 8.9E-02 | 5.9E-01 | 794.92 | 560.5 | 159.83 | **hsa-mir-107-A** |  |
| **215** | 9.2E-02 | 5.9E-01 | 4.93 | 15.84 | 23.31 | **hsa-mir-509-P** |  |
| **220** | 9.5E-02 | 6.0E-01 | 88.53 | 79.92 | 21.84 | **hsa-mir-370-A** |  |
| **222** | 9.6E-02 | 6.0E-01 | 7.46 | 2.1 | 9.49 | **hsa-mir-133a2-A** |  |
| **225** | 9.8E-02 | 6.0E-01 | 2630.83 | 2783.71 | 735.02 | **hsa-mir-342-A** |  |
| **226** | 9.8E-02 | 6.0E-01 | 2.82 | 3.12 | 17.57 | **hsa-mir-802-P** |  |
| **241** | 1.1E-01 | 6.2E-01 | 11.71 | 5.75 | 27.67 | **hsa-mir-516-4-3p-A** |  |
| **252** | 1.2E-01 | 6.4E-01 | 5.32 | 10.56 | 15.64 | **hsa-mir-123-P** |  |
| **259** | 1.2E-01 | 6.4E-01 | 18.58 | 10.2 | 44.13 | **hsa-mir-329-1-A** |  |
| **298** | 1.4E-01 | 6.5E-01 | 1209.08 | 874.69 | 410.17 | **hsa-mir-191-5p-A** |  |
| **314** | 1.5E-01 | 6.5E-01 | 30.52 | 66.99 | 21.99 | **hsa-mir-299-5p-A** |  |
| **316** | 1.5E-01 | 6.5E-01 | 595.58 | 710.07 | 294.32 | **hsa-mir-484-A** |  |
| **310** | 1.5E-01 | 6.5E-01 | 11.13 | 3.27 | 7.89 | **hsa-mir-520f-P** |  |
| **307** | 1.4E-01 | 6.5E-01 | 4.08 | 3.65 | 13.21 | **hsa-mir-617-A** |  |
| **313** | 1.5E-01 | 6.5E-01 | 16.17 | 5.1 | 20.99 | **hsa-mir-623-P** |  |
| **324** | 1.5E-01 | 6.5E-01 | 23.32 | 23.95 | 10.46 | **hsa-mir-511-1-P** |  |
| **323** | 1.5E-01 | 6.5E-01 | 13.96 | 7.25 | 21.93 | **hsa-mir-570-P** |  |
| **325** | 1.5E-01 | 6.6E-01 | 9.65 | 9.11 | 28.92 | **hsa-mir-521-2-A** |  |
| **328** | 1.6E-01 | 6.7E-01 | 16.35 | 4.37 | 11.2 | **hsa-mir-649-A** |  |
| **330** | 1.6E-01 | 6.7E-01 | 117.62 | 117.31 | 49.62 | **hsa-mir-125b1-A** |  |
| **336** | 1.6E-01 | 6.7E-01 | 4.32 | 6.27 | 1.85 | **hsa-mir-569-P** |  |
| **342** | 1.7E-01 | 6.7E-01 | 474.49 | 464.32 | 161.68 | **hsa-mir-594-P** |  |
| **362** | 1.8E-01 | 6.8E-01 | 6.98 | 19.23 | 23.75 | **hsa-mir-409-5p-A** |  |
| **347** | 1.7E-01 | 6.8E-01 | 3.41 | 1.35 | 15.77 | **hsa-mir-514-2&3-P** |  |
| **367** | 1.8E-01 | 6.8E-01 | 5.19 | 1.24 | 3.41 | **hsa-mir-582-A** |  |
| **379** | 1.9E-01 | 6.9E-01 | 25.63 | 49.51 | 12.44 | **hsa-mir-338-P** |  |
| **385** | 1.9E-01 | 6.9E-01 | 9.41 | 8.66 | 23.4 | **hsa-mir-519e-3p-A** |  |
| **411** | 2.1E-01 | 7.1E-01 | 2.01 | 2.3 | 8.07 | **hsa-mir-597-P** |  |
| **426** | 2.2E-01 | 7.2E-01 | 1.31 | 3.38 | 3.3 | **hsa-mir-302c-3p-A** |  |
| **433** | 2.2E-01 | 7.2E-01 | 14.88 | 4.83 | 5.07 | **hsa-mir-619-A** |  |
| **425** | 2.2E-01 | 7.2E-01 | 11.89 | 5.94 | 21.44 | **hsa-mir-653-A** |  |
| **444** | 2.3E-01 | 7.2E-01 | 1.13 | 1.37 | 3.35 | **hsa-mir-101-2-P** |  |
| **440** | 2.3E-01 | 7.2E-01 | 5.65 | 6.9 | 13.23 | **hsa-mir-21-P** |  |
| **451** | 2.3E-01 | 7.2E-01 | 13.39 | 5.45 | 11.23 | **hsa-mir-507-P** |  |
| **456** | 2.4E-01 | 7.2E-01 | 6.18 | 2.14 | 8.93 | **hsa-mir-509-A** |  |
| **486** | 2.5E-01 | 7.3E-01 | 21.98 | 5.39 | 13.72 | **hsa-mir-329-2-A** |  |
| **483** | 2.5E-01 | 7.3E-01 | 418.29 | 1602.8 | 950.9 | **hsa-mir-494-A** |  |
| **481** | 2.5E-01 | 7.3E-01 | 24.41 | 10.19 | 7.32 | **hsa-mir-521-1-A** |  |
| **479** | 2.5E-01 | 7.3E-01 | 3.55 | 12.19 | 13.93 | **hsa-mir-554-A** |  |
| **470** | 2.5E-01 | 7.3E-01 | 56.55 | 27.49 | 13.44 | **hsa-mir-583-P** |  |
| **491** | 2.6E-01 | 7.3E-01 | 84.22 | 150.63 | 52.9 | **hsa-mir-219-1-A** |  |
| **499** | 2.6E-01 | 7.4E-01 | 7.38 | 20.25 | 6.35 | **hsa-mir-133b-A** |  |
| **496** | 2.6E-01 | 7.4E-01 | 1.72 | 8.86 | 3.37 | **hsa-mir-369-3p-A** |  |
| **500** | 2.6E-01 | 7.4E-01 | 18.39 | 4.02 | 5.65 | **hsa-mir-559-P** |  |
| **515** | 2.7E-01 | 7.4E-01 | 4.7 | 2.28 | 1.78 | **hsa-mir-301-A** |  |
| **519** | 2.8E-01 | 7.5E-01 | 13.67 | 5.54 | 19.46 | **hsa-mir-576-P** |  |
| **554** | 3.0E-01 | 7.6E-01 | 6.4 | 2.71 | 10.21 | **hsa-mir-549-P** |  |
| **567** | 3.1E-01 | 7.7E-01 | 14.13 | 4.22 | 11.36 | **hsa-mir-362-A** |  |
| **565** | 3.1E-01 | 7.7E-01 | 4.3 | 4.03 | 8.51 | **hsa-mir-623-A** |  |
| **601** | 3.3E-01 | 7.7E-01 | 9.47 | 6.8 | 19.74 | **hsa_mir_490_Hcd20 right** | |
| **585** | 3.3E-01 | 7.7E-01 | 60.34 | 20.91 | 27.92 | **hsa-mir-32-A** |  |
| **589** | 3.3E-01 | 7.7E-01 | 4.41 | 1.7 | 6.09 | **hsa-mir-372-P** |  |
| **591** | 3.3E-01 | 7.7E-01 | 6.35 | 1.9 | 10.62 | **hsa-mir-517*c-5p-A** |  |
| **573** | 3.2E-01 | 7.7E-01 | 3.81 | 8.48 | 12.78 | **hsa-mir-567-A** |  |
| **574** | 3.2E-01 | 7.7E-01 | 10.25 | 8.09 | 3.8 | **hsa-mir-646-A** |  |
| **624** | 3.5E-01 | 7.9E-01 | 9.02 | 8.85 | 3.62 | **hsa-mir-101-1/2-P** |  |
| **623** | 3.5E-01 | 7.9E-01 | 5.99 | 11.82 | 20.37 | **hsa-mir-624-P** |  |
| **634** | 3.6E-01 | 8.0E-01 | 2.77 | 8.29 | 10.56 | **hsa-mir-519a1-5p/526c-A** | |
| **635** | 3.6E-01 | 8.0E-01 | 694.16 | 593.38 | 344.21 | **hsa-mir-92b-P** |  |
| **638** | 3.7E-01 | 8.0E-01 | 21.55 | 27.18 | 7.11 | **hsa-mir-296-P** |  |
| **647** | 3.7E-01 | 8.0E-01 | 8.01 | 15.66 | 20.4 | **hsa-mir-527-P** |  |
| **649** | 3.8E-01 | 8.1E-01 | 4.14 | 5.82 | 13.28 | **hsa-mir-526a-2-A** |  |
| **652** | 3.8E-01 | 8.1E-01 | 49.11 | 45.22 | 16.15 | **hsa-mir-30e-5p-A** |  |
| **661** | 3.9E-01 | 8.1E-01 | 2.8 | 4.99 | 7.43 | **hsa-mir-101-1/2-A** |  |
| **664** | 3.9E-01 | 8.1E-01 | 2.19 | 4.28 | 9.33 | **hsa-mir-380-5p-A** |  |
| **680** | 4.0E-01 | 8.3E-01 | 6.46 | 10.19 | 3.49 | **hsa-mir-219-2-A** |  |
| **673** | 4.0E-01 | 8.3E-01 | 5.37 | 8.43 | 21.47 | **hsa-mir-597-A** |  |
| **689** | 4.1E-01 | 8.3E-01 | 10.36 | 6.73 | 21.93 | **hsa-mir-135a-2-P** |  |
| **691** | 4.1E-01 | 8.3E-01 | 10.78 | 4.47 | 5.9 | **hsa-mir-511-2-A** |  |
| **686** | 4.1E-01 | 8.3E-01 | 7.35 | 4.27 | 12.34 | **hsa-mir-518f*-5p-A** |  |
| **699** | 4.2E-01 | 8.3E-01 | 34.23 | 54.44 | 19.05 | **hsa-mir-340-P** |  |
| **700** | 4.2E-01 | 8.3E-01 | 151.06 | 75.61 | 152.91 | **hsa-mir-34b-P** |  |
| **697** | 4.2E-01 | 8.3E-01 | 2.46 | 6.83 | 2.62 | **hsa-mir-519b-3p/526c-A** | |
| **709** | 4.3E-01 | 8.3E-01 | 1954.6 | 2055.35 | 960.53 | **hsa-mir-663-P** |  |
| **728** | 4.4E-01 | 8.4E-01 | 11.92 | 4.01 | 10.53 | **hsa-mir-588-P** |  |
| **725** | 4.4E-01 | 8.4E-01 | 34.42 | 5.96 | 22.82 | **hsa-mir-639-A** |  |
| **729** | 4.4E-01 | 8.5E-01 | 20.74 | 19.24 | 8.95 | **hsa-mir-577-P** |  |
| **738** | 4.5E-01 | 8.5E-01 | 56.63 | 132.78 | 55.35 | **hsa-mir-339-A** |  |
| **749** | 4.6E-01 | 8.6E-01 | 5.9 | 6.83 | 15.7 | **hsa-mir-154*-3p-A** |  |
| **752** | 4.6E-01 | 8.6E-01 | 370.9 | 370.03 | 157.63 | **hsa-mir-560-P** |  |
| **756** | 4.7E-01 | 8.6E-01 | 5.18 | 12.57 | 12.7 | **hsa-mir-20b-P** |  |
| **755** | 4.7E-01 | 8.6E-01 | 2.43 | 6.4 | 4.33 | **hsa-mir-488-P** |  |
| **763** | 4.7E-01 | 8.6E-01 | 16.74 | 8.91 | 23.82 | **hsa-miR-302c*-5p-A** |  |
| **777** | 4.8E-01 | 8.7E-01 | 5.17 | 10.61 | 10.31 | **hsa-mir-520d*-5p-A** |  |
| **780** | 4.9E-01 | 8.7E-01 | 212.57 | 397.98 | 149.43 | **hsa-miR-126*-5p-A** |  |
| **824** | 5.2E-01 | 8.8E-01 | 3.12 | 4.16 | 2.29 | **hsa-mir-100-P** |  |
| **803** | 5.1E-01 | 8.8E-01 | 8.53 | 26.98 | 20.34 | **hsa-mir-431-A** |  |
| **818** | 5.2E-01 | 8.8E-01 | 16.31 | 7.91 | 8.99 | **hsa-mir-513-2-A** |  |
| **799** | 5.0E-01 | 8.8E-01 | 9.32 | 3.03 | 8.7 | **hsa-mir-518f-3p-A** |  |
| **821** | 5.2E-01 | 8.8E-01 | 6.78 | 11.14 | 20.97 | **hsa-mir-524-3p-A** |  |
| **801** | 5.0E-01 | 8.8E-01 | 7.46 | 6.97 | 15.79 | **hsa-mir-555-A** |  |
| **849** | 5.4E-01 | 8.8E-01 | 2.85 | 7.24 | 4.63 | **hsa-mir-591-A** |  |
| **842** | 5.3E-01 | 8.8E-01 | 7.02 | 8.15 | 11.81 | **hsa-miR-767-5p-A** |  |
| **877** | 5.6E-01 | 8.9E-01 | 18.66 | 10.26 | 10.66 | **hsa-mir-582-P** |  |
| **880** | 5.7E-01 | 8.9E-01 | 10.79 | 6.29 | 12.43 | **hsa-mir-606-P** |  |
| **873** | 5.6E-01 | 8.9E-01 | 4.12 | 1.64 | 2.62 | **hsa-mir-633-A** |  |
| **878** | 5.6E-01 | 8.9E-01 | 1.28 | 3.06 | 2.62 | **hsa-mir-98-A** |  |
| **893** | 5.8E-01 | 9.0E-01 | 9.35 | 6.69 | 4.34 | **hsa-mir-544-P** |  |
| **888** | 5.7E-01 | 9.0E-01 | 9.32 | 5.13 | 7.96 | **hsa-mir-626-A** |  |
| **887** | 5.7E-01 | 9.0E-01 | 10.82 | 6.82 | 4.78 | **hsa-mir-645-P** |  |
| **909** | 6.0E-01 | 9.1E-01 | 6.62 | 11.12 | 12.59 | **hsa-mir-758-A** |  |
| **914** | 6.0E-01 | 9.2E-01 | 14.68 | 21.96 | 13.49 | **hsa-mir-127-A** |  |
| **913** | 6.0E-01 | 9.2E-01 | 3.48 | 7.87 | 2.71 | **hsa-miR-302c-3p** |  |
| **916** | 6.1E-01 | 9.2E-01 | 10.1 | 10.82 | 20.24 | **hsa-mir-520b-P** |  |
| **926** | 6.1E-01 | 9.2E-01 | 13.35 | 13.04 | 23.35 | **hsa-mir-520h-P** |  |
| **925** | 6.1E-01 | 9.2E-01 | 3.52 | 1.63 | 3.65 | **hsa-mir-571-A** |  |
| **938** | 6.2E-01 | 9.2E-01 | 12.66 | 9.81 | 6.05 | **hsa-mir-628-A** |  |
| **947** | 6.3E-01 | 9.2E-01 | 6.01 | 9.67 | 13.02 | **hsa-mir-627-P** |  |
| **955** | 6.4E-01 | 9.3E-01 | 14.22 | 7.9 | 11.22 | **hsa-mir-628-P** |  |
| **994** | 6.6E-01 | 9.3E-01 | 6.57 | 10.33 | 4.12 | **hsa-mir-29b-1-P** |  |
| **980** | 6.6E-01 | 9.3E-01 | 5.03 | 2.99 | 3.11 | **hsa-mir-302b-3p-A** |  |
| **991** | 6.6E-01 | 9.3E-01 | 1.82 | 2.32 | 4.39 | **hsa-mir-340-A** |  |
| **988** | 6.6E-01 | 9.3E-01 | 2.14 | 2.33 | 3.94 | **hsa-mir-612-P** |  |
| **997** | 6.7E-01 | 9.3E-01 | 5.27 | 5.79 | 3.15 | **hsa-mir-368-A** |  |
| **999** | 6.7E-01 | 9.3E-01 | 9.69 | 12.42 | 4.96 | **hsa-mir-99a-A** |  |
| **1008** | 6.8E-01 | 9.4E-01 | 15.8 | 9.11 | 16.7 | **hsa-mir-452*-3p-A** |  |
| **1017** | 6.9E-01 | 9.5E-01 | 6.37 | 9.13 | 10.18 | **hsa_mir_490_Hcd20 left** | |
| **1013** | 6.9E-01 | 9.5E-01 | 7.53 | 3.94 | 4.23 | **hsa-mir-513-1-A** |  |
| **1027** | 7.0E-01 | 9.5E-01 | 3.39 | 4 | 7.66 | **hsa-mir-610-A** |  |
| **1045** | 7.2E-01 | 9.5E-01 | 5.02 | 2.81 | 4.06 | **hsa-mir-101-1-P** |  |
| **1048** | 7.2E-01 | 9.5E-01 | 11.52 | 18.93 | 16.49 | **hsa-mir-19b2-A** |  |
| **1099** | 7.6E-01 | 9.6E-01 | 12.51 | 15.28 | 21.46 | **hsa-mir-134-A** |  |
| **1074** | 7.4E-01 | 9.6E-01 | 6.4 | 11.61 | 7.46 | **hsa-mir-187-P** |  |
| **1097** | 7.6E-01 | 9.6E-01 | 21.24 | 13.87 | 14.85 | **hsa-mir-18b-A** |  |
| **1062** | 7.4E-01 | 9.6E-01 | 17.65 | 30.16 | 21.25 | **hsa-mir-196a-2-P** |  |
| **1104** | 7.6E-01 | 9.6E-01 | 8.64 | 11.04 | 16.59 | **hsa-mir-224-A** |  |
| **1094** | 7.6E-01 | 9.6E-01 | 44.22 | 32.52 | 52.17 | **hsa-mir-33b-A** |  |
| **1075** | 7.4E-01 | 9.6E-01 | 2.28 | 3.26 | 1.82 | **hsa-mir-367-P** |  |
| **1108** | 7.6E-01 | 9.6E-01 | 5.05 | 5.75 | 7.81 | **hsa-mir-384-A** |  |
| **1063** | 7.4E-01 | 9.6E-01 | 3.68 | 3.66 | 6.67 | **hsa-mir-506-P** |  |
| **1107** | 7.6E-01 | 9.6E-01 | 12.55 | 12.11 | 17.67 | **hsa-mir-518a2-3p-A** |  |
| **1081** | 7.5E-01 | 9.6E-01 | 12.93 | 14.74 | 8.1 | **hsa-mir-520c-3p-A** |  |
| **1109** | 7.7E-01 | 9.6E-01 | 4.98 | 9.28 | 6.08 | **hsa-mir-520g-A** |  |
| **1076** | 7.4E-01 | 9.6E-01 | 4.07 | 4.5 | 8.19 | **hsa-mir-559-A** |  |
| **1088** | 7.5E-01 | 9.6E-01 | 2.7 | 4.08 | 6.39 | **hsa-mir-586-P** |  |
| **1121** | 7.8E-01 | 9.7E-01 | 7.93 | 9.29 | 15.53 | **hsa-mir-421-P** |  |
| **1122** | 7.8E-01 | 9.7E-01 | 13.46 | 8.61 | 10.44 | **hsa-mir-501-A** |  |
| **1125** | 7.8E-01 | 9.7E-01 | 2.23 | 1.59 | 2.8 | **hsa-mir-580-A** |  |
| **1129** | 7.9E-01 | 9.7E-01 | 15.85 | 15.27 | 11.26 | **hsa-mir-448-P** |  |
| **1150** | 8.1E-01 | 9.7E-01 | 7.54 | 11.45 | 9.32 | **hsa-mir-376a2-P** |  |
| **1167** | 8.2E-01 | 9.7E-01 | 21.02 | 22.31 | 13.25 | **hsa-mir-548a2-A** |  |
| **1144** | 8.0E-01 | 9.7E-01 | 14.45 | 9.28 | 10.38 | **hsa-mir-605-P** |  |
| **1176** | 8.2E-01 | 9.7E-01 | 11.35 | 13.5 | 19.13 | **hsa-mir-96-P** |  |
| **1194** | 8.4E-01 | 9.8E-01 | 2.85 | 3.8 | 2.75 | **hsa-mir-368-P** |  |
| **1191** | 8.3E-01 | 9.8E-01 | 13.47 | 20.01 | 14.35 | **hsa-mir-510-A** |  |
| **1178** | 8.3E-01 | 9.8E-01 | 3.3 | 5.7 | 5.87 | **hsa-mir-606-A** |  |
| **1183** | 8.3E-01 | 9.8E-01 | 11.79 | 9.27 | 12.77 | **hsa-mir-652-P** |  |
| **1211** | 8.5E-01 | 9.8E-01 | 117.05 | 86.74 | 82.07 | **hsa_mir_147 left** |  |
| **1196** | 8.4E-01 | 9.8E-01 | 3.52 | 4.5 | 7.78 | **hsa-mir-191*-3p-A** |  |
| **1206** | 8.5E-01 | 9.8E-01 | 4.19 | 5.68 | 4.04 | **hsa-mir-592-P** |  |
| **1208** | 8.5E-01 | 9.8E-01 | 8.43 | 12.52 | 8.77 | **hsa-mir-614-A** |  |
| **1205** | 8.5E-01 | 9.8E-01 | 6.96 | 12 | 10.08 | **hsa-mir-624-A** |  |
| **1223** | 8.6E-01 | 9.8E-01 | 83.09 | 67.54 | 60.68 | **hsa-mir-146b-P** |  |
| **1219** | 8.6E-01 | 9.8E-01 | 2.61 | 1.73 | 1.82 | **hsa-mir-374-P** |  |
| **1232** | 8.7E-01 | 9.8E-01 | 10.65 | 10.48 | 7.51 | **hsa-mir-432-5p-A** |  |
| **1220** | 8.6E-01 | 9.8E-01 | 15.36 | 11.42 | 11.49 | **hsa-mir-512-1&2-3p-A** | |
| **1228** | 8.7E-01 | 9.8E-01 | 8.96 | 5.79 | 7.06 | **hsa-mir-649-P** |  |
| **1225** | 8.6E-01 | 9.8E-01 | 5.09 | 7.2 | 6.53 | **hsa-mir-802-A** |  |
| **1259** | 8.9E-01 | 9.8E-01 | 6.98 | 7.68 | 10.32 | **hsa-mir-522-5p/526c-A** | |
| **1274** | 9.0E-01 | 9.8E-01 | 65.78 | 68.69 | 48.85 | **hsa-mir-26a-1-P** |  |
| **1284** | 9.1E-01 | 9.9E-01 | 6.35 | 6.25 | 9.04 | **hsa-mir-196b-A** |  |
| **1306** | 9.3E-01 | 9.9E-01 | 13.38 | 12.18 | 9.35 | **hsa-mir-133a1-A** |  |
| **1316** | 9.4E-01 | 9.9E-01 | 5.87 | 4.38 | 4.06 | **hsa-mir-30e-3p-A** |  |
| **1311** | 9.3E-01 | 9.9E-01 | 7.79 | 6.01 | 7.59 | **hsa-mir-620-A** |  |
| **1322** | 9.4E-01 | 9.9E-01 | 11.49 | 13.34 | 13.58 | **hsa-mir-148a-A** |  |
| **1324** | 9.4E-01 | 9.9E-01 | 10.35 | 8.42 | 7.42 | **hsa-mir-450-1-A** |  |
| **1320** | 9.4E-01 | 9.9E-01 | 10.3 | 9.32 | 7.92 | **hsa-mir-539-A** |  |
| **1321** | 9.4E-01 | 9.9E-01 | 6.18 | 6.07 | 4.69 | **hsa-mir-618-P** |  |
| **1325** | 9.4E-01 | 9.9E-01 | 42.51 | 51.63 | 59.62 | **hsa-mir-622-A** |  |
| **1335** | 9.5E-01 | 9.9E-01 | 3.98 | 5.59 | 4.19 | **hsa-mir-614-P** |  |
| **1358** | 9.7E-01 | 9.9E-01 | 8.45 | 9.31 | 7.53 | **hsa-mir-337-P** |  |
| **1348** | 9.6E-01 | 9.9E-01 | 11.84 | 14.49 | 11.19 | **hsa-mir-556-A** |  |
| **1372** | 9.8E-01 | 1.0E+00 | 13.95 | 14 | 12.31 | **hsa-mir-153-2-A** |  |
| **1371** | 9.8E-01 | 1.0E+00 | 7.72 | 9.29 | 8.64 | **hsa-mir-651-P** |  |
| **1377** | 9.9E-01 | 1.0E+00 | 3.75 | 4.08 | 3.34 | **hsa-mir-34b-A** |  |
|  |  |  |  |  |  |  |  |

## Supplementary Table 2. putative targets of miR-30 after WGE analysis of miR-30 family KD and Pre-miR-30a. Potential targets common to the first WGE analysis (miR-30a modulation) are underlined.

|  |  |  |  |  |  |
| --- | --- | --- | --- | --- | --- |
| **Gene symbol** | **miR-159 KD** | **miR-30 family KD** | **Pre-miR-30a** | **p-value** | **FDR** |
|  |  |  |  |  |  |
|  |  |  |  |  |  |
| ***ABCA12*** | 83.11 | 126.97 | 61.11 | 4.90E-06 | 0.01 |
| ***ACAT2*** | 1567.59 | 1709.60 | 1154.67 | 1.71E-04 | 0.06 |
| ***ACTR1A*** | 758.37 | 893.57 | 532.00 | 1.97E-04 | 0.06 |
| ***AFF3*** | 191.59 | 216.96 | 105.33 | 1.30E-06 | 0.00 |
| ***AGR2*** | 128.77 | 170.46 | 85.76 | 1.20E-05 | 0.01 |
| ***ALG10B*** | 93.28 | 103.20 | 73.56 | 8.41E-04 | 0.13 |
| ***AP3S1*** | 718.70 | 779.04 | 452.75 | 5.92E-04 | 0.12 |
| ***ATG12*** | 827.96 | 1029.21 | 566.93 | 4.72E-04 | 0.11 |
| ***ATG12*** | 123.60 | 144.99 | 97.65 | 7.38E-04 | 0.13 |
| ***ATP6V1B2*** | 968.73 | 1049.54 | 653.29 | 2.94E-05 | 0.02 |
| ***AVEN*** | 206.19 | 243.27 | 116.20 | 9.50E-06 | 0.01 |
| ***B4GALT4*** | 187.77 | 216.12 | 125.29 | 5.39E-05 | 0.03 |
| ***BNIP3L*** | 193.56 | 217.97 | 122.55 | 4.60E-06 | 0.01 |
| ***BTBD10*** | 510.60 | 520.12 | 346.00 | 1.65E-04 | 0.06 |
| ***C14orf129*** | 487.44 | 557.92 | 324.08 | 2.60E-06 | 0.01 |
| ***C1orf174*** | 444.22 | 517.86 | 310.17 | 8.54E-05 | 0.04 |
| ***C1orf19*** | 472.52 | 557.77 | 301.84 | 3.20E-06 | 0.01 |
| ***C2orf30*** | 447.45 | 534.21 | 250.24 | 1.20E-06 | 0.00 |
| ***C3orf57*** | 863.83 | 903.80 | 341.05 | 2.00E-07 | 0.00 |
| ***CASP3*** | 214.70 | 255.12 | 141.63 | 1.62E-04 | 0.06 |
| ***CBFB*** | 336.90 | 362.57 | 225.43 | 2.68E-04 | 0.08 |
| ***CBLB*** | 170.70 | 191.03 | 115.71 | 8.53E-05 | 0.04 |
| ***CBX2*** | 1688.19 | 1857.51 | 1155.49 | 2.67E-04 | 0.08 |
| ***CENPH*** | 171.61 | 178.07 | 120.23 | 7.17E-04 | 0.12 |
| ***CHMP2B*** | 529.70 | 560.41 | 374.87 | 2.19E-04 | 0.07 |
| ***CLDND1*** | 468.39 | 497.34 | 359.94 | 3.23E-04 | 0.08 |
| ***CPSF2*** | 302.51 | 363.96 | 181.15 | 8.30E-06 | 0.01 |
| ***CPSF2*** | 353.02 | 403.43 | 212.23 | 7.59E-05 | 0.04 |
| ***CREG1*** | 631.61 | 657.83 | 472.37 | 8.53E-04 | 0.14 |
| ***CYB561*** | 1779.34 | 2007.18 | 1288.69 | 6.18E-04 | 0.12 |
| ***CYB561*** | 1750.51 | 1766.28 | 1215.33 | 9.80E-04 | 0.14 |
| ***DCUN1D3*** | 243.46 | 264.62 | 159.39 | 1.06E-05 | 0.01 |
| ***DDIT4*** | 690.54 | 856.96 | 487.23 | 3.23E-05 | 0.02 |
| ***DGUOK*** | 1757.00 | 2025.53 | 1322.86 | 9.62E-04 | 0.14 |
| ***DLX1*** | 288.23 | 362.52 | 181.09 | 2.21E-05 | 0.02 |
| ***DPY19L1*** | 394.53 | 440.63 | 218.63 | 4.60E-06 | 0.01 |
| ***DPYSL2*** | 764.95 | 828.74 | 441.69 | 3.85E-05 | 0.02 |
| ***ELMOD2*** | 160.47 | 181.41 | 120.52 | 9.74E-05 | 0.04 |
| ***EML1*** | 222.25 | 255.99 | 141.32 | 4.80E-05 | 0.03 |
| ***EXTL2*** | 158.60 | 176.59 | 111.57 | 5.70E-04 | 0.12 |
| ***FAM18B*** | 333.75 | 340.42 | 244.32 | 5.04E-04 | 0.11 |
| ***FAM18B*** | 573.60 | 596.01 | 403.41 | 5.20E-04 | 0.11 |
| ***FAM43A*** | 97.64 | 123.15 | 69.94 | 3.80E-06 | 0.01 |
| ***FBXL20*** | 120.22 | 125.57 | 86.47 | 5.23E-04 | 0.11 |
| ***FBXO45*** | 226.13 | 238.85 | 142.49 | 2.57E-04 | 0.08 |
| ***FOXD1*** | 318.94 | 328.75 | 180.92 | 3.80E-06 | 0.01 |
| ***GLCE*** | 291.64 | 331.54 | 187.71 | 6.58E-05 | 0.04 |
| ***GNAI2*** | 728.17 | 934.79 | 386.32 | 2.00E-07 | 0.00 |
| ***GNPDA1*** | 1451.66 | 1622.29 | 880.79 | 6.00E-07 | 0.00 |
| ***GSTM1*** | 210.10 | 246.15 | 134.21 | 2.10E-05 | 0.02 |
| ***GSTM1*** | 511.16 | 560.89 | 305.25 | 2.36E-05 | 0.02 |
| ***GTF2E2*** | 1206.48 | 1409.14 | 811.47 | 1.08E-04 | 0.05 |
| ***HDGFRP3*** | 200.15 | 244.41 | 163.87 | 1.73E-04 | 0.06 |
| ***ICK*** | 320.24 | 352.96 | 235.47 | 4.87E-04 | 0.11 |
| ***IDH1*** | 791.52 | 1088.07 | 348.35 | < 1e-07 | < 1e-07 |
| ***IDH1*** | 369.51 | 417.18 | 153.45 | < 1e-07 | < 1e-07 |
| ***IRX4*** | 257.67 | 311.77 | 175.46 | 9.64E-04 | 0.14 |
| ***KCTD5*** | 1584.03 | 1648.44 | 1112.22 | 7.50E-04 | 0.13 |
| ***KDELC2*** | 232.15 | 243.19 | 128.84 | 4.20E-06 | 0.01 |
| ***KIAA1279*** | 433.27 | 449.57 | 316.06 | 8.75E-04 | 0.14 |
| ***KLHDC2*** | 565.49 | 625.73 | 397.76 | 4.93E-04 | 0.11 |
| ***MARCKS*** | 643.78 | 795.84 | 439.30 | 2.24E-04 | 0.07 |
| ***MPHOSPH6*** | 215.01 | 235.99 | 162.76 | 2.03E-04 | 0.07 |
| ***NDEL1*** | 648.72 | 778.39 | 464.07 | 3.24E-04 | 0.08 |
| ***NECAP1*** | 470.29 | 554.08 | 325.66 | 2.27E-05 | 0.02 |
| ***NEDD4*** | 285.48 | 320.85 | 190.50 | 1.83E-05 | 0.02 |
| ***NR2F2*** | 291.39 | 317.41 | 191.49 | 4.55E-04 | 0.11 |
| ***NRP1*** | 339.99 | 409.09 | 249.40 | 5.33E-04 | 0.11 |
| ***NSMAF*** | 292.50 | 357.19 | 230.06 | 8.68E-04 | 0.14 |
| ***P4HA2*** | 375.48 | 465.55 | 283.36 | 1.98E-04 | 0.06 |
| ***PCGF6*** | 385.45 | 402.19 | 285.46 | 8.26E-04 | 0.13 |
| ***PDSS1*** | 797.43 | 912.33 | 585.97 | 3.39E-04 | 0.09 |
| ***PDSS1*** | 394.23 | 448.04 | 296.98 | 5.22E-04 | 0.11 |
| ***PGM1*** | 491.32 | 645.13 | 329.31 | 4.00E-06 | 0.01 |
| ***PGM3*** | 269.04 | 325.01 | 178.45 | 1.53E-05 | 0.01 |
| ***PGM3*** | 221.92 | 230.57 | 152.30 | 6.03E-04 | 0.12 |
| ***PIK3R2*** | 2560.86 | 2963.42 | 1523.11 | 2.90E-06 | 0.01 |
| ***PIP4K2A*** | 644.45 | 667.48 | 296.22 | 4.00E-07 | 0.00 |
| ***PPIL3*** | 1320.66 | 1593.93 | 930.74 | 2.20E-04 | 0.07 |
| ***PPP1R2*** | 992.47 | 1260.03 | 658.85 | 3.80E-06 | 0.01 |
| ***PPP2R1B*** | 129.29 | 147.19 | 101.49 | 2.09E-04 | 0.07 |
| ***PPP3CA*** | 174.97 | 191.48 | 119.47 | 1.44E-04 | 0.05 |
| ***PTGFRN*** | 235.67 | 273.27 | 140.03 | 1.45E-05 | 0.01 |
| ***PTGFRN*** | 127.88 | 136.04 | 85.04 | 9.69E-05 | 0.04 |
| ***RAB32*** | 335.19 | 364.98 | 239.75 | 2.60E-04 | 0.08 |
| ***RASA1*** | 670.15 | 737.60 | 463.83 | 2.92E-04 | 0.08 |
| ***REEP1*** | 260.96 | 304.88 | 152.64 | 1.97E-05 | 0.02 |
| ***RELL1*** | 147.14 | 154.74 | 98.35 | 1.37E-05 | 0.01 |
| ***RPA2*** | 1023.36 | 1136.76 | 743.45 | 5.96E-04 | 0.12 |
| ***RTN4R*** | 118.98 | 143.14 | 87.37 | 3.37E-04 | 0.09 |
| ***SAP30*** | 258.79 | 288.51 | 181.72 | 1.74E-04 | 0.06 |
| ***SBF1*** | 457.34 | 558.09 | 242.61 | 3.02E-05 | 0.02 |
| ***SBK1*** | 203.65 | 216.22 | 138.71 | 4.62E-04 | 0.11 |
| ***SCML1*** | 309.55 | 310.19 | 200.38 | 3.97E-04 | 0.10 |
| ***SEC23A*** | 185.09 | 197.20 | 96.44 | 6.00E-07 | 0.00 |
| ***SFXN1*** | 617.05 | 738.95 | 408.30 | 6.41E-04 | 0.12 |
| ***SLC29A3*** | 300.99 | 366.44 | 207.43 | 9.19E-05 | 0.04 |
| ***SLC35A5*** | 176.84 | 224.46 | 143.23 | 3.90E-04 | 0.10 |
| ***SNX6*** | 295.40 | 332.48 | 230.17 | 5.89E-04 | 0.12 |
| ***SSBP2*** | 461.31 | 537.64 | 360.64 | 6.30E-04 | 0.12 |
| ***ST3GAL5*** | 153.64 | 181.13 | 119.62 | 3.04E-04 | 0.08 |
| ***ST8SIA4*** | 114.36 | 121.34 | 90.35 | 6.78E-04 | 0.12 |
| ***ST8SIA4*** | 94.55 | 108.98 | 76.01 | 7.13E-04 | 0.12 |
| ***TASP1*** | 202.75 | 217.67 | 152.93 | 1.57E-04 | 0.06 |
| ***TBCC*** | 347.82 | 389.71 | 240.97 | 1.78E-04 | 0.06 |
| ***TBPL1*** | 470.72 | 531.55 | 304.25 | 1.35E-04 | 0.05 |
| ***TDG*** | 2138.54 | 2626.21 | 1578.27 | 3.47E-05 | 0.02 |
| ***TFDP1*** | 1431.67 | 1488.89 | 893.01 | 8.44E-05 | 0.04 |
| ***TFDP1*** | 1405.91 | 1470.61 | 903.55 | 1.47E-04 | 0.05 |
| ***TM4SF1*** | 1044.16 | 1062.78 | 674.68 | 8.10E-06 | 0.01 |
| ***TMEM69*** | 465.21 | 469.93 | 309.16 | 1.12E-05 | 0.01 |
| ***TNRC15*** | 230.22 | 252.74 | 164.27 | 8.93E-04 | 0.14 |
| ***TRIM37*** | 2878.87 | 2978.17 | 1798.53 | 3.04E-05 | 0.02 |
| ***TSEN2*** | 524.86 | 609.25 | 371.25 | 3.96E-04 | 0.10 |
| ***TXNDC5*** | 1208.07 | 1302.28 | 745.94 | 1.18E-05 | 0.01 |
| ***UBE2F*** | 325.20 | 330.79 | 233.74 | 5.68E-04 | 0.12 |
| ***UBN1*** | 1046.12 | 1090.12 | 756.98 | 3.34E-04 | 0.09 |
| ***VAMP3*** | 629.52 | 735.78 | 450.44 | 5.81E-04 | 0.12 |
|  |  |  |  |  |  |
