# Supplementary material for: MicroRNA miR-30 family regulates non-attachment growth of breast cancer cells
Source: BMC Genomics. 2013 Feb 28;14:139. doi: 10.1186/1471-2164-14-139 (PMC3602027; doi:10.1186/1471-2164-14-139)
Supplement: Additional file 6 — 420 gene sets sorted by LS permutation p-value (significant p-values are in red). Table of Gene Sets: 42 gene sets sorted by LS permutation p-value (significant p-values are in red). Table of Gene Sets: 43 gene sets sorted by LS permutation p-value (significant p-values are in red). [file 1471-2164-14-139-S6.pdf]

Type of Gene Sets: Gene Ontology

Number of total investigated Gene Sets: 4365

Type of univariate test used: F-test

Random variance model was used.

Tests used to find significant gene sets are: LS/KS permutation test, Efron-Tibshirani's GSA maxmean test

The threshold of determining significant gene sets is 0.005

LS/KS permutation test finds gene sets which have more genes differentially expressed among the phenotype classes than expected by chance.

Efron-Tibshirani's test uses 'maxmean' statistics to identify gene sets differentially expressed.

Summary of Results:

420 out of 4365 investigated gene sets passed the 0.005 significance threshold

LS/KS permutation test found 417 significant gene sets.

Efron-Tibshirani's maxmean test found 68 significant gene sets (under 200 permutations).

Table - Table of Gene Sets: 420 gene sets sorted by LS permutation p-value (significant p-values are in red)

	GO category	GO term	Number ofgenes	LS permutation p-value	KS permutation p-value	Efron-Tibshirani's GSA testp-value
1	GO:0000910	cytokinesis	87	0.00001	0.00002	0.025
5	GO:0006081	cellular aldehyde metabolic process	48	0.00001	0.00005	< 0.005
6	GO:0006084	acetyl-CoA metabolic process	57	0.00001	0.00001	< 0.005
7	GO:0006103	2-oxoglutarate metabolic process	22	0.00001	0.00146	< 0.005
11	GO:0006487	protein amino acid N-linked glycosylation	56	0.00001	0.00001	< 0.005
12	GO:0006733	oxidoreduction coenzyme metabolic process	73	0.00001	0.00001	< 0.005
14	GO:0006740	NADPH regeneration	17	0.00001	0.0037	< 0.005
17	GO:0006984	ER-nucleus signaling pathway	45	0.00001	0.00001	0.005
19	GO:0008334	histone mRNA metabolic process	33	0.00001	0.00065	0.005
20	GO:0009060	aerobic respiration	53	0.00001	0.00001	< 0.005
21	GO:0009109	coenzyme catabolic process	38	0.00001	0.00001	< 0.005
23	GO:0009311	oligosaccharide metabolic process	51	0.00001	0.00001	< 0.005
25	GO:0019362	pyridine nucleotide metabolic process	59	0.00001	0.00021	< 0.005

28	GO:0032288	myelin assembly	9	0.00001	0.00001	< 0.005
29	GO:0043558	regulation of translational initiation in response to stress	10	0.00001	0.00132	< 0.005
30	GO:0043648	dicarboxylic acid metabolic process	66	0.00001	0.00007	0.005
33	GO:0046356	acetyl-CoA catabolic process	34	0.00001	0.00001	< 0.005
34	GO:0046487	glyoxylate metabolic process	6	0.00001	0.00149	< 0.005
35	GO:0046496	nicotinamide nucleotide metabolic process	55	0.00001	0.00027	< 0.005
36	GO:0051187	cofactor catabolic process	44	0.00001	0.00001	< 0.005
44	GO:0004448	isocitrate dehydrogenase activity	9	0.00001	0.00006	< 0.005
50	GO:0016860	intramolecular oxidoreductase activity	53	0.00001	0.0005	< 0.005
53	GO:0051287	NAD or NADH binding	74	0.00001	0.00001	< 0.005
56	GO:0009312	oligosaccharide biosynthetic process	22	0.00001	0.00097	< 0.005
58	GO:0042375	quinone cofactor metabolic process	21	0.00003	0.00073	0.005
67	GO:0034969	histone arginine methylation	7	0.00006	0.00008	< 0.005
113	GO:0008023	transcription elongation factor complex	23	0.0003	0.00012	< 0.005
138	GO:0018216	peptidyl-arginine methylation	6	0.0005	0.00001	< 0.005
139	GO:0018195	peptidyl-arginine modification	9	0.0005	0.00001	< 0.005
144	GO:0033059	cellular pigmentation	38	0.00056	0.0015	0.005
156	GO:0035246	peptidyl-arginine N-methylation	5	0.00068	0.00007	0.005
186	GO:0009296	flagellum assembly	10	0.00119	0.00016	0.005
187	GO:0043064	flagellum organization	10	0.00119	0.00016	0.005
197	GO:0003352	regulation of cilium movement	6	0.00147	0.00028	0.005
198	GO:0060294	cilium movement involved in cell motility	6	0.00147	0.00028	0.005
199	GO:0060295	regulation of cilium movement involved in cell motility	6	0.00147	0.00028	0.005
205	GO:0032933	SREBP-mediated signaling pathway	8	0.00163	0.00001	0.005
289	GO:0003711	transcription elongation regulator activity	26	0.00368	0.0013	0.005

Type of Gene Sets: Biocarta Pathway

Number of total investigated Gene Sets: 307

Type of univariate test used: F-test

Random variance model was used.

Summary of Results:

42 out of 307 investigated gene sets passed the 0.005 significance threshold

LS/KS permutation test found 42 significant gene sets.

Efron-Tibshirani's maxmean test found 3 significant gene sets (under 200 permutations).

Table - Table of Gene Sets: 42 gene sets sorted by LS permutation p-value (significant p-values are in red)

	Biocarta Pathway	Pathway description	Number ofgenes	LS permutation p-value	KS permutation p-value	Efron-Tibshirani's GSA testp-value
1	h_cpsfPathway	Polyadenylation of mRNA	17	0.00001	0.0002	0.005
17	h_egfPathway	EGF Signaling Pathway	52	0.0014	0.00085	< 0.005

Type of Gene Sets: Kegg Pathway

Number of total investigated Gene Sets: 123

Type of univariate test used: F-test

Random variance model was used.

Summary of Results:

43 out of 123 investigated gene sets passed the 0.005 significance threshold

LS/KS permutation test found 43 significant gene sets.

Efron-Tibshirani's maxmean test found 3 significant gene sets (under 200 permutations).

Table - Table of Gene Sets: 43 gene sets sorted by LS permutation p-value (significant p-values are in red)

To access the list of genes within each gene set, click the hyperlinked number of genes for each gene set.

	Kegg Pathway	Pathway description	Number of genes	LS permutation p-value	KS permutation p-value	Efron-Tibshirani's GSA testp-value
3	hsa00052	Galactose metabolism	63	0.00001	0.00019	0.005
10	hsa00720	Reductive carboxylate cycle (CO2 fixation)	18	0.00001	0.00001	< 0.005
